# Interlocked 2D Covalent Organic Frameworks from Overcrowded Nodes

**DOI:** 10.1021/jacs.4c14453

**Published:** 2025-01-13

**Authors:** Elisabet De Bolòs, Saibal Bera, Karol Strutyński, Andrei A. Bardin, Rhys W. Lodge, Natalia M Padial, Akinori Saeki, Carlos Martí-Gastaldo, Andrei N. Khlobystov, Brent L. Nannenga, Manuel Melle-Franco, Aurelio Mateo-Alonso

**Affiliations:** † POLYMAT, University of the Basque Country UPV/EHU, Avenida de Tolosa 72, Donostia-San Sebastián 20018, Spain; ‡ CICECO—Aveiro Institute of Materials, Department of Chemistry, University of Aveiro, Aveiro 3810-193, Portugal; § Chemical Engineering, School for Engineering of Matter, Transport, and Energy, 7864 Arizona State University, Tempe, Arizona 85287, United States; ∥ Center for Applied Structural Discovery, The Biodesign Institute, Arizona State University, Tempe, Arizona 85281, United States; ⊥ School of Chemistry, 6123 University of Nottingham, University Park, Nottingham NG7 2RD, United Kingdom; # The Nanoscale and Microscale Research Centre, University of Nottingham, University Park, Nottingham NG7 2RD, United Kingdom; ∇ Instituto de Ciencia Molecular, 16781 Universidad de Valencia, Paterna 46980, Spain; ○ Department of Applied Chemistry, Graduate School of Engineering, 13013 Osaka University, Suita, Osaka 565-0871, Japan; ◆ Ikerbasque, Basque Foundation for Science, Bilbao 48013, Spain

## Abstract

A challenging aspect in the synthesis of covalent organic frameworks (COFs) that goes beyond the framework’s structure and topology is interpenetration, where two or more independent frameworks are mechanically interlocked with each other. Such interpenetrated or interlocked frameworks are commonly found in three-dimensional (3D) COFs with large pores. However, interlocked two-dimensional (2D) COFs are rarely seen in the literature, as 2D COF layers typically crystallize in stacks that maximize stabilization through π-stacking. The few interlocked 2D COFs described to date have been derived from monomers with aryl groups arranged perpendicularly. Herein, we report an interlocked 2D COF derived from a new class of monomers constituted of sterically overcrowded polycyclic aromatic hydrocarbons. The formation of such an interlocked structure is ascribed to the presence and the bulkiness of the substituents that directly interfere with interlayer π-stacking. The microscopy, gas sorption, spectroscopic, and charge transport characterization are consistent with the absence of π-stacking, as imposed by the interlocked architecture. This work evidences how the use of overcrowded aromatic systems as monomers can generate mechanically interlocked 2D COFs, offering new avenues for the design of COFs with unconventional topologies.

## Graphical abstract

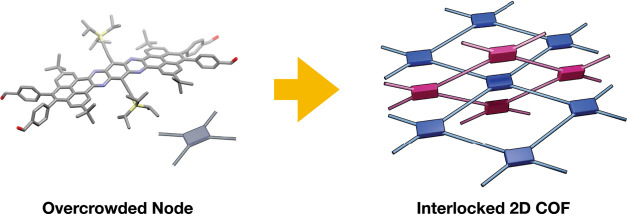

## Introduction

Covalent organic frameworks (COFs) are crystalline polymeric two-dimensional (2D) and three-dimensional (3D) structures constituted of covalently linked organic monomers.^1–7^ The crystal structure and the properties of COFs are directly related to the symmetry, connectivity, and nature of the monomers. Therefore, the rational design of monomers has become a fundamental tool in the design of COFs, as it allows the blueprinting and synthesizing of COFs with specific structures, topologies, and properties. Yet, a challenging aspect in the synthesis of COFs that goes beyond the framework’s structure and topology is interpenetration. In an interpenetrated COF, two or more independent frameworks are mechanically interlocked with each other.^2,4,8–11^ Such interpenetrated or interlocked frameworks are commonly found in 3D COFs with large pores, within which additional nets can intergrow. Interpenetration reduces the void space and surface area of 3D COFs while strengthening internetwork interactions, which significantly enhances the stability and charge transport properties of 3D COFs.^2,4,8–10^ However, interlocked 2D COFs are rarely found in the literature,^12–14^ as 2D COF layers typically crystallize in stacks that maximize stabilization through π-stacking. The few interlocked 2D COFs described to date have been derived from monomers featuring aryl groups arranged perpendicularly in the core,^12–14^ either via a quaternary (spiro) carbon^12^ or due to restricted rotation (Figure 1).^13,14^ This orthogonal arrangement of the monomer core’s aryl groups, when incorporated into 2D COFs, prevents interlayer π-stacking, thereby favoring the formation of interlocked structures.

**Figure 1:**
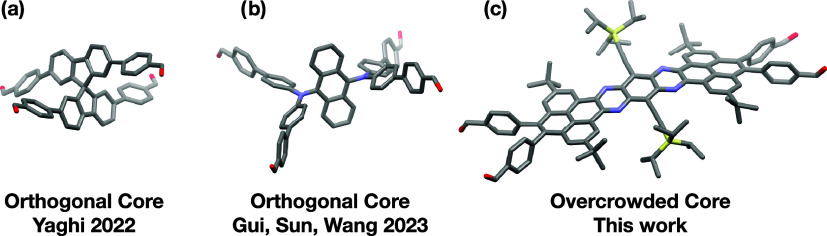
Selected monomer designs that generate interlocked 2D and 3D COFs.

Herein, we report an interlocked 2D COF (**Bet-COF-1**) derived from a new class of monomers constituted of sterically overcrowded polycyclic aromatic hydrocarbons (Figures 1 and 2a). Monomer **1** is constituted by a tetrabenzotetraazaheptacene core functionalized with 4-formylphenyl, *tert*-butyl, and tri*iso*-propylsilylacetylene (TIPS-acetylene) substituents. The condensation of monomer **1** with *p*-phenylenediamine (**2**) through imine bonds generates a single-pore rhombic framework that crystallizes in an interlocked fashion with an inclined interpenetrated topology (Figure 2). The formation of such an interlocked structure is ascribed to the presence and bulkiness of the *tert*-butyl and TIPS-acetylene substituents present on the tetrabenzotetraazaheptacene nodes that interfere with interlayer π-stacking. The crystal structure of **Bet-COF-1** has been solved by a combination of microcrystal electron diffraction (MicroED) and theoretical calculations and is consistent with spectroscopic, microscopy, and gas sorption data. Ultaviolet–visible-near infrared (UV–vis-NIR) absorption, fluorescence, and charge transport studies combined with theoretical investigations show how the observed optoelectronic properties are consistent with the absence of π-stacking, as imposed by the interlocked architecture.

**Figure 2:**
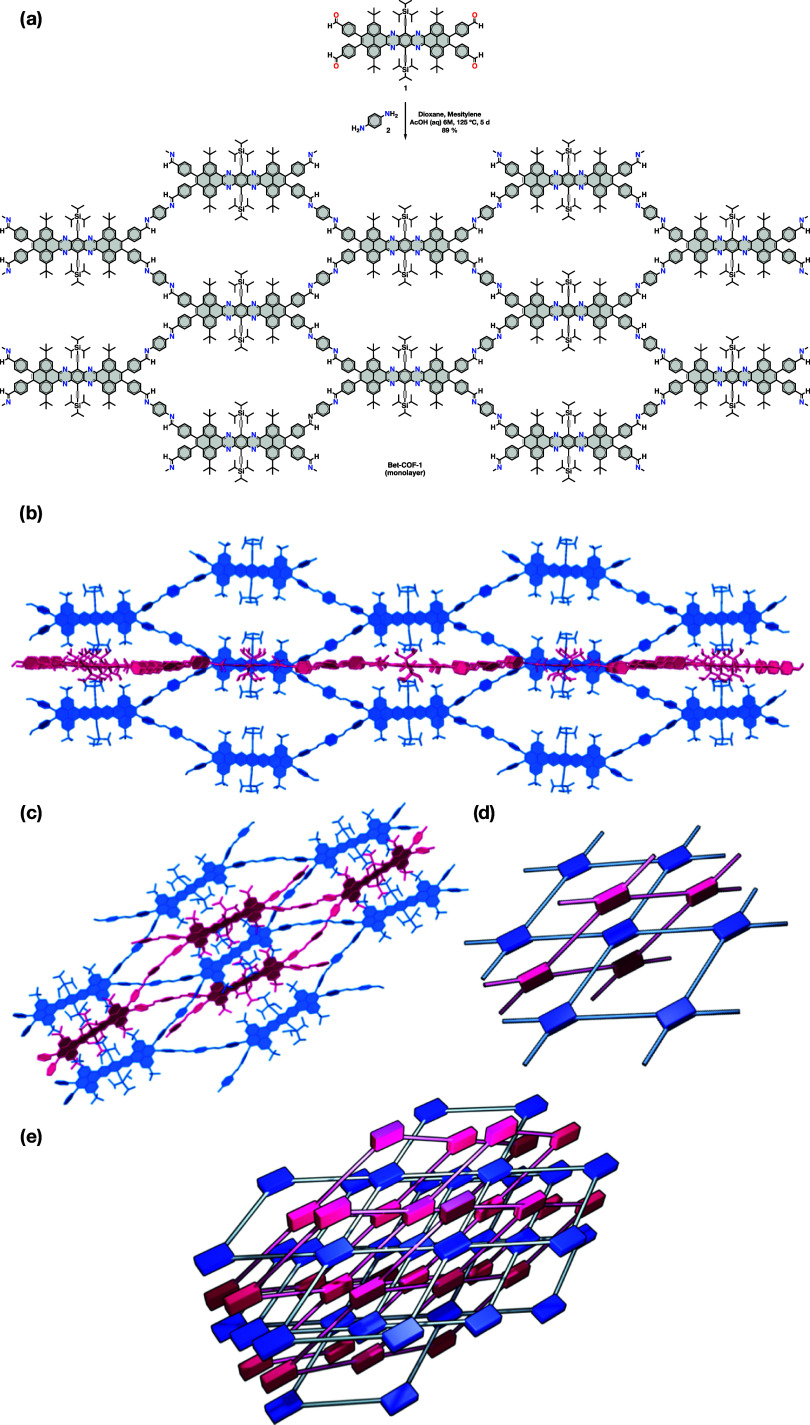
(a) Front and (b) side views of a single layer of **Bet-COF-1** in the crystal structure, (c–e) different views of the crystal structure of **Bet-COF-1** illustrating the interpenetrated architecture. Hydrogens were omitted for clarity.

We selected pyrene-fused azaacene derivatives as nodes because of their synthetic accessibility and enhanced stability.^15^ Tetrabenzotetraazaheptacene **1** was synthesized in two steps from 2,7-di-*tert*-butylpyrene-9,10-dibromo-4,5-tetraone (**3**) and 1,2,4,5-tetraamino-3,6-bis-[(tri*iso*-propylsilyl)­ethynyl]-benzene (**4**)^16^ (Scheme 1), which were respectively obtained in three and five steps from commercially available compounds (details are presented in Supporting Information). First, condensation of 2,7-di-*tert*-butylpyrene-9,10-dibromo-4,5-tetraone **3** with tetramine (**4**) in chloroform and acetic acid yielded tetrabromotetrabenzoheptacene **5** (29%) as a purple powder that was sufficiently soluble to be characterized by ^1^H NMR and matrix-assisted laser desorption/ionization time-of-flight mass spectrometry (MALDI-TOF MS). Then, Suzuki coupling between tetrabromotetrabenzoheptacene **5** and 4-formylphenylboronic acid (**6**) yielded tetrabenzotetraazaheptacene **1** (32%) as a purple powder that showed optimal solubility for solution synthesis. The structure of **1** was unambiguously confirmed by ^1^H NMR, ^13^C NMR, MALDI-MS, and single-crystal X-ray diffraction (SCXRD). The crystal structure of compound **1** reveals the overcrowded and nearly planar tetrabenzotetraazaheptacene core that adopts a slightly distorted conformation with the TIPS-acetylene groups bending outward from the aromatic plane (Scheme 1). These small distortions are reflected by the end-to-center and pyrene-to-acetylene dihedral angles of 11 and 12°, respectively, and are consistent with the proximity and the steric demand of the *tert*-butyl and TIPS-acetylene groups and with previous observations on pyrene-fused twistacenes.^17–21^

**Scheme 1:**
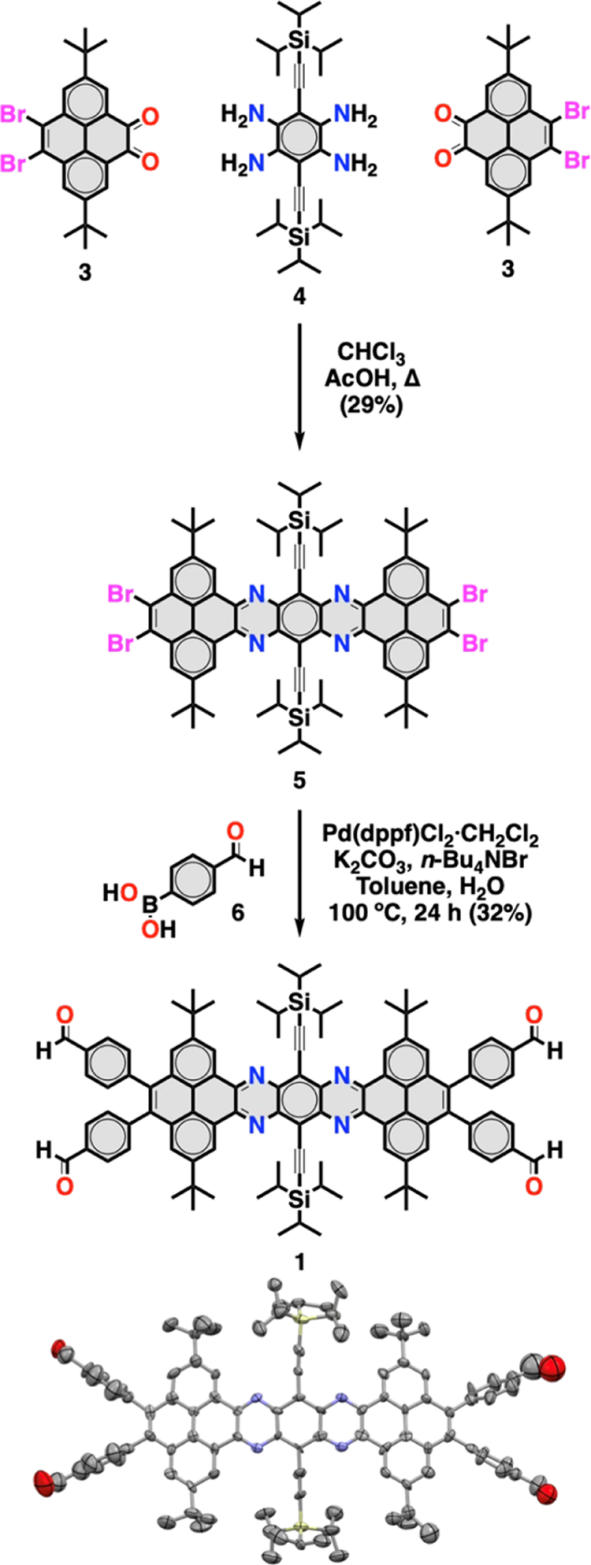
Synthesis and Crystal Structure of Monomer **1**

**Bet-COF-1** was synthesized solvothermally by reacting tetrabenzotetraazaheptacene **1** with *p*-phenylenediamine (**2**) in a mixture of dioxane and mesitylene (1:1) and in the presence of a 6 M aqueous solution of acetic acid. The reaction mixture was sealed in an ampule and held at 125 °C for 5 days. Then, the **Bet-COF-1** solids were isolated and washed by filtration. The powder XRD (PXRD) pattern of **Bet-COF-1** shows many sharp, well-resolved X-ray reflections (Figures 3a and S1). The high crystallinity is also confirmed by high resolution transmission electron microscopy (HRTEM) that shows uniform rounded-corner rod-shaped microcrystals with lengths between 0.5 and 1 μm, a width of ∼200 nm, and a periodic lattice in the microcrystals (Figures 3b–d and S2). Energy-dispersive X-ray spectroscopy (EDX) performed on individual nanocrystals demonstrates the presence of Si and N peaks as well as C (Figure S2). The structural and spectroscopic characterization is consistent with the formation of a 2D COF. For instance, the Fourier transform infrared (FTIR) spectrum of **Bet-COF-1** shows the imine C=N stretch band (1626 cm^–1^) and the attenuation of the aldehyde C=O stretch band (1712 cm^–1^) and of the amine bands (3372 cm^–1^) found, respectively, in the spectra of precursors **1** and **2** (Figure 3e). This is in agreement with the cross-polarization/magic angle spinning (CP/MAS) solid-state ^13^C nuclear magnetic resonance (NMR) spectrum of **Bet-COF-1** that shows exclusively a resonance peak at 165 ppm for the carbon of the C=N bond corresponding to the imine bond and does not show signs of the resonance aldehyde peak at 192 ppm present in the spectrum of monomer **1** (Figure 3f). Additionally, the NMR spectrum of the hydrolysis/digestion of the crystalline powder **Bet-COF-1** in TFA-*d*_1_ shows the signals of the hydrolyzed tetrabenzotetraazaheptacene **1** and *p*-phenylenediamine (**2**) monomers in a 1:2 ratio (Figure 3g), in agreement with the 1:2 stoichiometry of **Bet-COF-1**.

**Figure 3:**
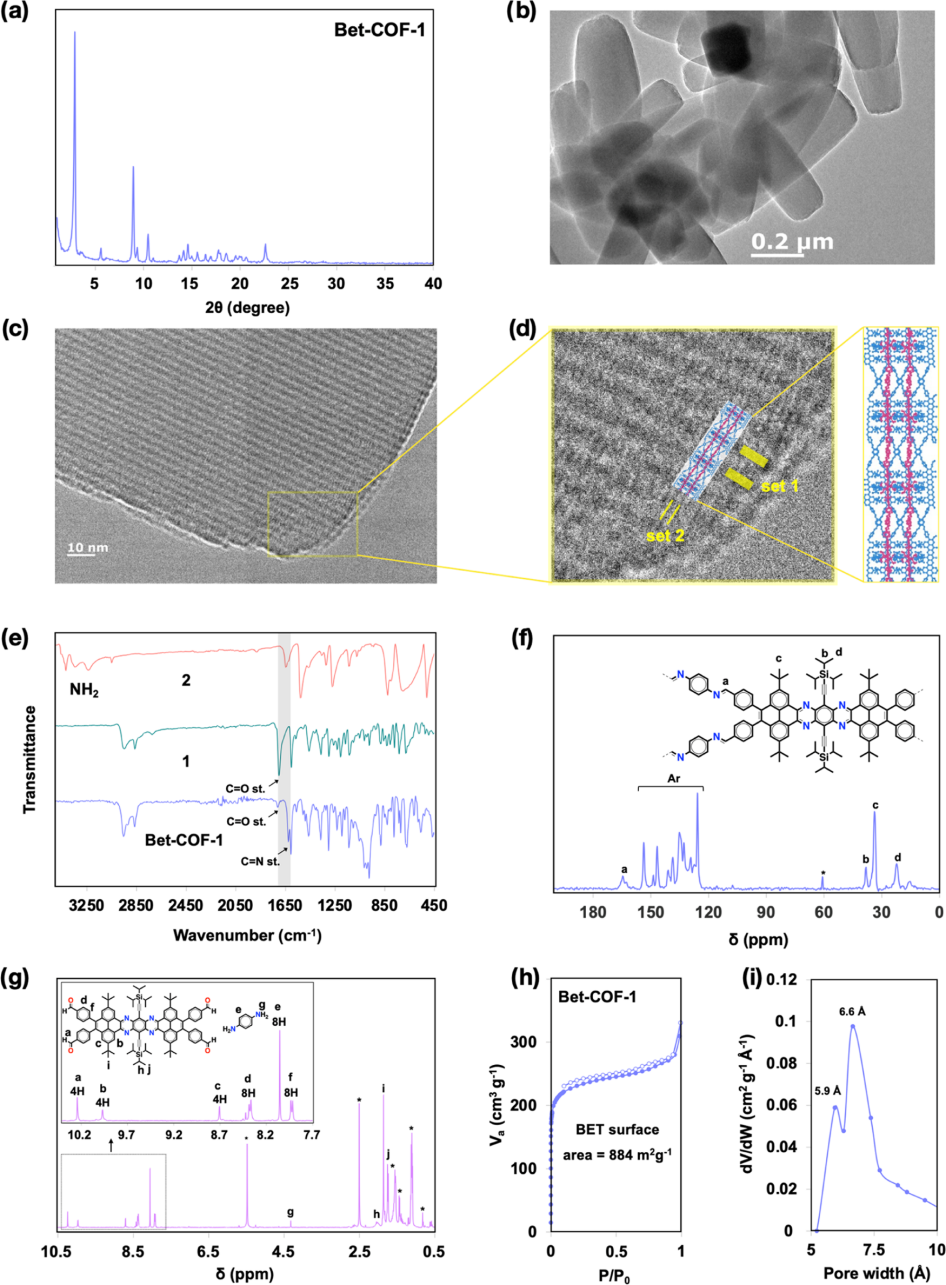
(a) PXRD pattern of **Bet-COF-1**. (b) HRTEM image of **Bet-COF-1**. (c) HRTEM image of an edge of a microcrystal of **Bet-COF-1** illustrating two sets of lattice fringes—wide, high contrast running parallel to the long edge and fine perpendicular. (d) Set 1 corresponds to a COF plane with tetrabenzotetraazaheptacene units within the plane of the image and set 2 to a plane with tetrabenzotetraazaheptacene units perpendicular to the plane of the image (structural diagram of **Bet-COF-1** within the (011) is overlaid on the HRTEM image). (e) FTIR spectra of tetrabenzotetraazaheptacene **1**, *p*-phenylenediamine (**2**) and **Bet-COF-1**. (f) SS CP-MAS ^13^C NMR spectrum of **Bet-COF-1**. (g) ^1^H NMR of a hydrolyzed **Bet-COF-1** sample in TFA-*d*_1_ showing the signals of tetrabenzotetraazaheptacene **1** and *p*-phenylenediamine (**2**) monomers (*indicates solvent residual peaks). (h) Nitrogen adsorption and desorption isotherm profiles at 77 K and (i) pore size distribution of **Bet-COF-1**.

The crystal structure of **Bet-COF-1** (Figure 1b–e) was solved by a combination of MicroED^22^ and theoretical calculations. MicroED was carried out with data from 2 crystals at a specimen temperature of ∼100 K that was collected and merged together to produce a refined structure in the orthorhombic *Pnc*2 space group. The structure showed the tetrabenzotetraazaheptacene nodes and their arrangement (*a* = 65.63 Å, *b* = 13.80 Å, *c* = 14.13 Å, *R*1= 18.42%, and w*R*2= 46.22%) (Table S1). The MicroED structure was completed with the missing linkers, and then molecular dynamics simulations with a fixed unit cell and a tight-binding Hamiltonian was performed to explore the conformational space thoroughly. Selected structures were optimized with Density Functional Theory (DFT) to produce the final 3D structure (see the SI for details). The theoretical PXRD pattern of **Bet-COF-1** shows a very good correlation with the experimental PXRD pattern (Figure S3). The powder pattern was better refined on the tetragonal variant of **Bet-COF-1** (*a* = *b* = 14.31 Å, *c* = 65.62 Å, *R*_wp_= 19.28, and *R*_exp_= 5.14) (Table S2). This suggests that even though the dominant phase is tetragonal and agrees well with the powder diffraction pattern of the bulk, we could find only nanocrystals with good electron diffraction in a lower symmetry. The crystal structure unambiguously confirms that **Bet-COF-1** is a 2D COF with a rhombic pore (sql), in which the tetrabenzotetraazaheptacene nodes are covalently bound to the *p*-phenylenediamine linkers by four imines (Figure 1b–e). The different 2D COF layers are interlocked with each other in an inclined interpenetration fashion, where the frameworks grow interlocked with each other practically perpendicularly. The TIPS groups of the nodes of one net sit perpendicularly on top of the node of the interlocked net in a herringbone arrangement (Figure 1d). A likely rationale for the formation of an interlocked architecture, rather than of a stacked architecture, is the presence and the bulkiness of the *tert*-butyl and TIPS-acetylene substituents present on the tetrabenzotetraazaheptacene nodes that interfere with interlayer π-stacking. This is consistent with the previous literature that illustrates how monomers with orthogonal cores that interfere with π-stacking generate interlocked 2D COFs,^12–14^ whereas planar-core monomers with 4-formylphenyl (or 4-aminophenyl) groups generate π-stacked 2D COFs.^23–28^

HRTEM imaging of individual microparticles of **Bet-COF-1** at high magnification reveals crystal lattice fringes consistent with the projection of the (011) plane (Figure 3c,d) shown in the structural diagram in Figure 2a. The high contrast areas forming 3 nm lattice fringes (set 1, Figure 3d) correspond to the columns of tetrabenzotetrazaheptacene moieties within the (011) plane. Perpendicular to the high contrast features, a set of finer 1 nm lattice fringes can be observed (set 2, Figure 3d) that correspond to the interlocked **Bet-COF-1** nets perpendicular to the (011) plane. The pores in the microcrystal appear as areas of lower contrast running parallel to the tetrabenzotetrazaheptacene columns.

The interlocked topology of **Bet-COF-1** lines up two different micropores within the space generated by the linkers, as evidenced by nitrogen sorption–desorption experiments performed at −196 °C after activation at 100 °C under a vacuum. At this temperature, **Bet-COF-1** is perfectly stable as thermal gravimetric analysis illustrates signs of decomposition >200 °C (Figure S4). The Brunauer–Emmett–Teller (BET) surface area of **Bet-COF-1** is 884 m^2^ g^–1^ (Figure 3h), which is consistent with the theoretical Poreblazer and Zeo++ surface areas calculated for the crystal structure of **Bet-COF-1** (863 and 870 m^2^ g^–1^, respectively). The experimental pore size distributions give two dominant pores of 5.9 and 6.6 Å (Figure 3i) that are consistent with the theoretical pore values of 5.6 and 6.4 Å generated by the space between the *p*-phenylenediimine linkers (Figure S5).

The optoelectronic properties are also consistent with the formation of an interlocked 2D COF. The solid-state UV–vis–NIR electronic absorption spectrum of **Bet-COF-1** shows an absorption pattern similar to that of tetrabenzotetraazaheptacene **1** (Figure 4a). The optical band gap of **Bet-COF-1** estimated according to the Kubelka–Munk-transformed reflectance spectrum corresponds to 1.99 eV (Figure 4b), which is in very good agreement with HOCO-LUCO gap of 2.01 eV, computed at the B3LYP/light level, and very similar to the values of 2.11 and 2.09 eV from the forming two separated monolayers, which are electronically uncoupled (Figures 4c and S6). The HOCOs show density on nodes (e.g., HOCO–1 and HOCO–3) and linkers (e.g., HOCO and HOCO–2) (Figure S7). The LUCO and LUCO+1, and LUCO+2 and LUCO+3 are nearly degenerated and localized on the nodes (Figure S8). Interestingly, there is a noticeable gap of 1.0 eV at the B3LYP level between LUCO+3 and LUCO+4, the latter of which is localized in the linkers (Figure S8). **Bet-COF-1** is fluorescent in the solid state, which is consistent with the absence of π-stacking, as observed in another 2D interlocked COF.^14^ Solid-state fluorescence spectroscopy evidence a fluorescence band centered in the NIR that spans from 650–850 nm, in agreement with the band observed for monomer **1** (Figure 4d), but less broad and red-shifted in the case of **Bet-COF-1**. Furthermore, a low maximum φ∑μ value (φ∑μ_max_) of 2.1 × 10^–5^ cm^2^ V^–1^ s^–1^ was obtained by flash-photolysis time-resolved microwave conductivity (FP-TRMC) (Figure 4e), which is consistent with the lack of π-stacking as a result of the interpenetration of the nets (typical φ∑μ_max_ values observed for π-stacked 2D COFs^29–40^ are in the order of 10^–4^ cm^2^ V^–1^ s^–1^).

**Figure 4:**
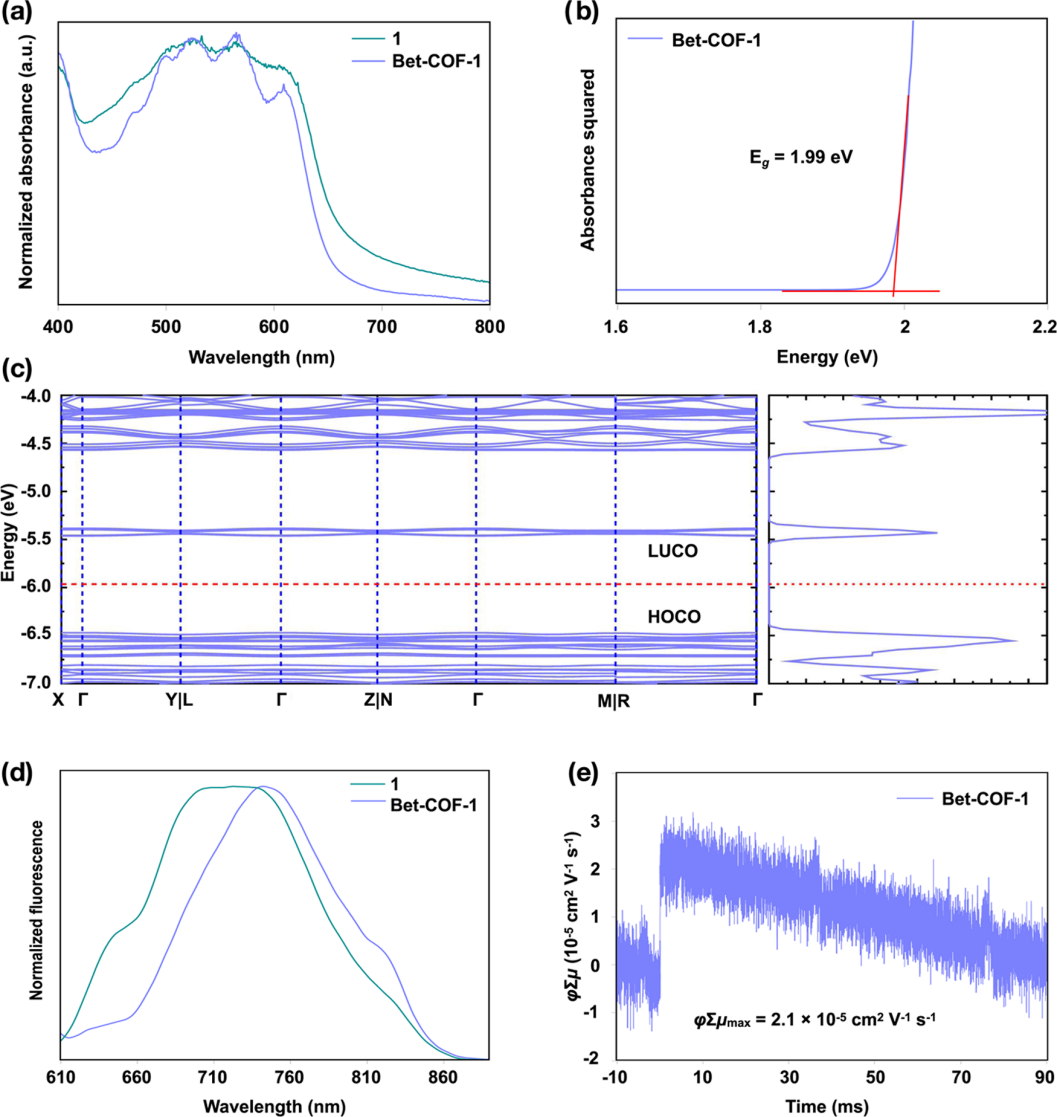
(a) Solid-state UV–vis-NIR absorption spectra of tetrabenzotetraazaheptacene **1** and **Bet-COF-1**. (b) Estimated optical band gap of **Bet-COF-1**. (c) Band structure (left) and corresponding density of states (right) of **BET-COF-1**. (d) Solid-state fluorescence spectra of tetrabenzotetraazaheptacene **1** and **Bet-COF-1**. (e) FP-TRMC conductivity transients of **Bet-COF-1** upon excitation at 355 nm, 9.1 × 10^15^ photons cm^–2^ pulse^–1^.

## Conclusions

We reported the synthesis of a novel interlocked 2D covalent organic framework (COF) derived from a new class of sterically congested monomers. The formation of this interlocked structure is attributed to the presence of bulky *tert*-butyl and TIPS-acetylene substituents on the monomers, which disrupt interlayer π-stacking during COF formation, favoring the observed interlocked architecture. The crystal structure of **Bet-COF-1** was determined using a combination of microcrystal electron diffraction and theoretical calculations, which are consistent with microscopy, gas sorption, and spectroscopic data. Additionally, the absence of π-stacking is reflected in the observed optoelectronic and charge transport properties, such as solid-state fluorescence and a φ∑μ_max_ value that is 1 order of magnitude lower than those typically observed in π-stacked 2D COFs. This work shows how the use of overcrowded aromatic systems as monomers can generate mechanically interlocked 2D COFs, offering new avenues for the design of COFs with unconventional topologies. Furthermore, it allows the synthesis of fluorescent COFs that may find application in light-emitting and sensing applications,^41,42^ among others.

## Supplementary Material



## Notes

Comparative PXRD patterns of monomers **1** and **2** and Bet-COF-1; Pawley refinement of **Bet-COF-1**; energies of frontier orbitals of **Bet-COF-**1; MicroED Data collection, processing and refinement statistics; summary of the parameters obtained from the Pawley refinement; materials, and methods (PDF)

## Abbreviations

COF: covalent organic framework
MicroED: microcrystal electron diffraction
NMR: nuclear magnetic resonance
MALDI-TOF MS: matrix-assisted laser desorption/ionization time-of-flight mass spectrometry
TIPS: tri*iso*-propylsilyl
PXRD: powder X-ray diffraction
EDX: energy-dispersive X-ray spectroscopy
HRTEM: high-resolution transmission electron microscopy
TFA: trifluoroacetic acid
BET surface area: Brunauer–Emmett–Teller surface area
HOCO: highest occupied crystal orbital
LUCO: highest unoccupied crystal orbital
FP-TRMC: flash-photolysis time-resolved microwave conductivity
