# Supplementary material for: Interlocked 2D Covalent Organic Frameworks from Overcrowded Nodes
Source: J Am Chem Soc. 2025 Jan 13;147(3):2579–86. doi: 10.1021/jacs.4c14453 (PMC12265044; doi:10.1021/jacs.4c14453)
Supplement: Supplementary file 1 [file ja4c14453_si_001.pdf]

Interlocked 2D Covalent Organic Frameworks from Overcrowded Nodes

Elisabet De Bolòs,[1] Saibal Bera,[1] Karol Strutyński,[2] Andrei A. Bardin,[3,4] Rhys W. Lodge,[5,6] Natalia M. Padial,[7] Akinori Saeki,[8] Carlos Martí-Gastaldo,[7] Andrei N. Khlobystov,[5,6] Brent L. Nannenga,[3,4] Manuel Melle-Franco,[2]* and Aurelio Mateo-Alonso[1,9]*

[1]*POLYMAT, University of the Basque Country UPV/EHU, Avenida de Tolosa 72, Donostia-San Sebastián 20018, Spain. E-mail: amateo@polymat.eu*

[2]*CICECO - Aveiro Institute of Materials, Department of Chemistry, University of Aveiro, Aveiro 3810-193, Portugal. E-mail: manuelmelle@ua.pt*

[3]*Chemical Engineering, School for Engineering of Matter, Transport, and Energy, Arizona State University, Tempe, Arizona 85287, USA*

[4]*Center for Applied Structural Discovery, The Biodesign Institute, Arizona State University, Tempe, Arizona 85281, USA*

[5]*School of Chemistry, University of Nottingham, University Park, Nottingham NG7 2RD, United Kingdom*

[6]*The Nanoscale and Microscale Research Centre, University of Nottingham, University Park, Nottingham NG7 2RD, United Kingdom*

[7]*Instituto de Ciencia Molecular, Universidad de Valencia, Paterna 46980, Spain*

[8]*Department of Applied Chemistry, Graduate School of Engineering, Osaka University, Suita, Osaka 565-0871, Japan*

[9]*Ikerbasque, Basque Foundation for Science, Bilbao 48013, Spain*

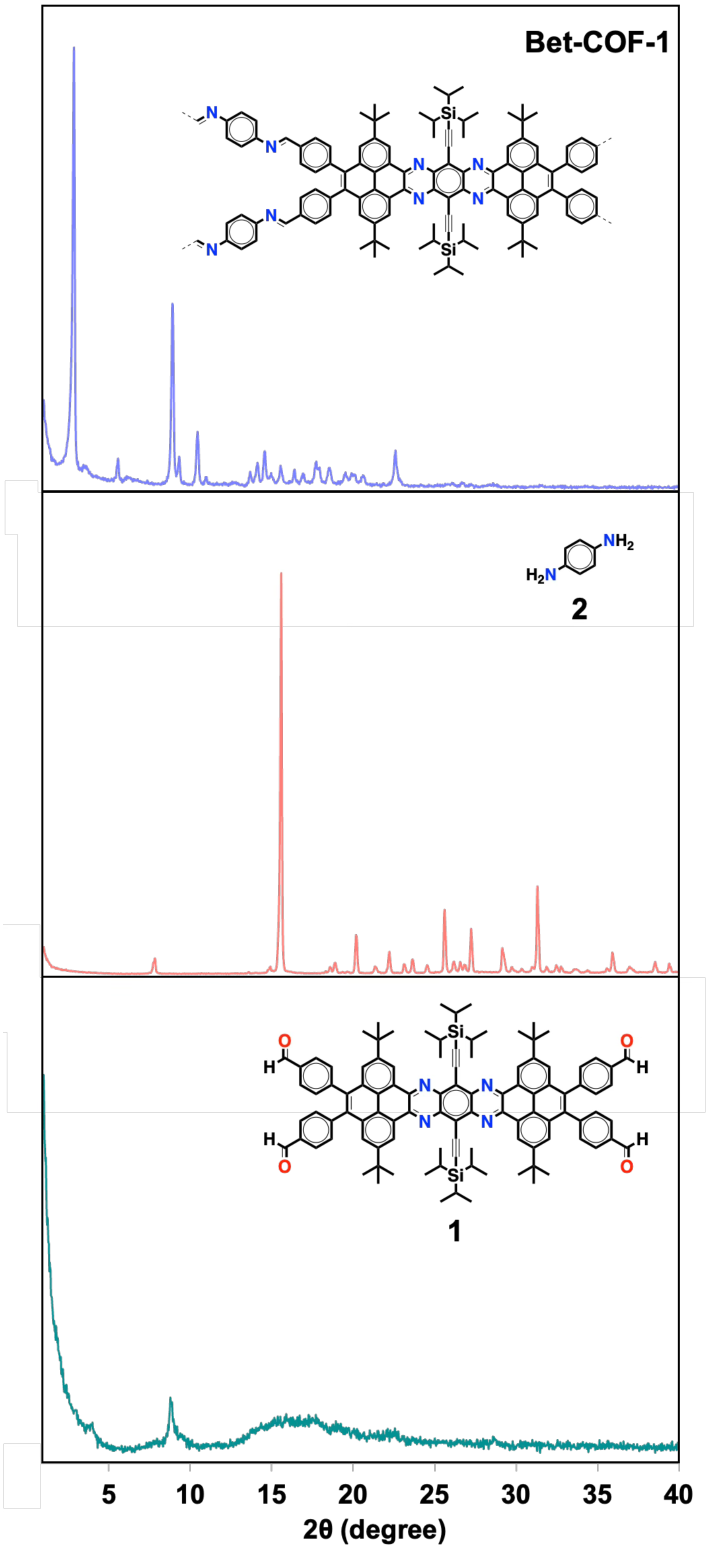

Figure S1. Comparative PXRD patterns of monomers **1** and **2** and **Bet-COF-1**.

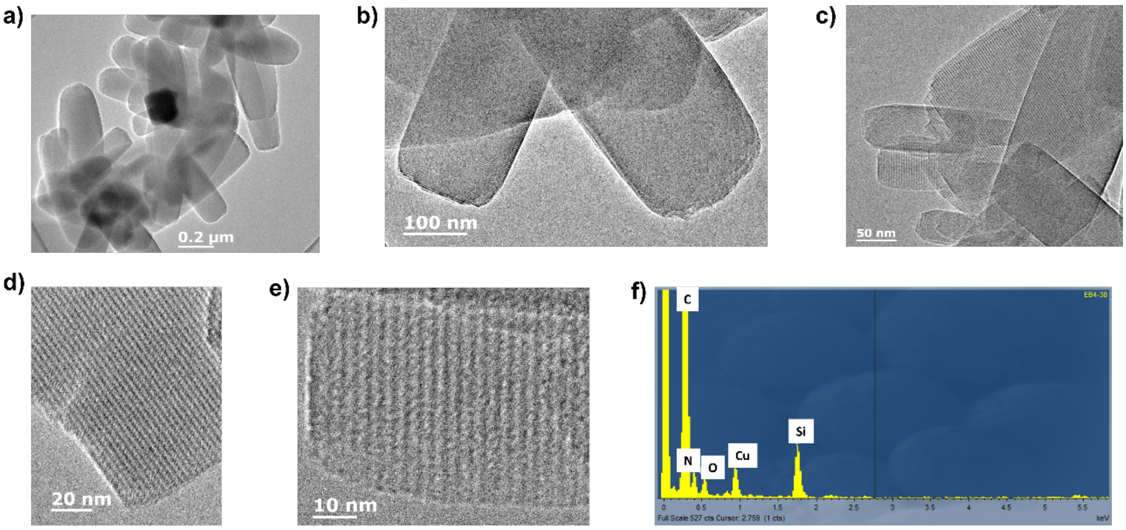

Figure S2. (a-c) HRTEM images of **Bet-COF-1** microcrystals illustrating. (d-e) High magnification HRTEM images showing lattice fringes corresponding to the positions of tetrabenzotetraazaheptacene units. (f) EDX spectrum of an individual nanocrystal showing the presence of carbon, silicon and nitrogen (Cu peak is from the TEM sample holder).

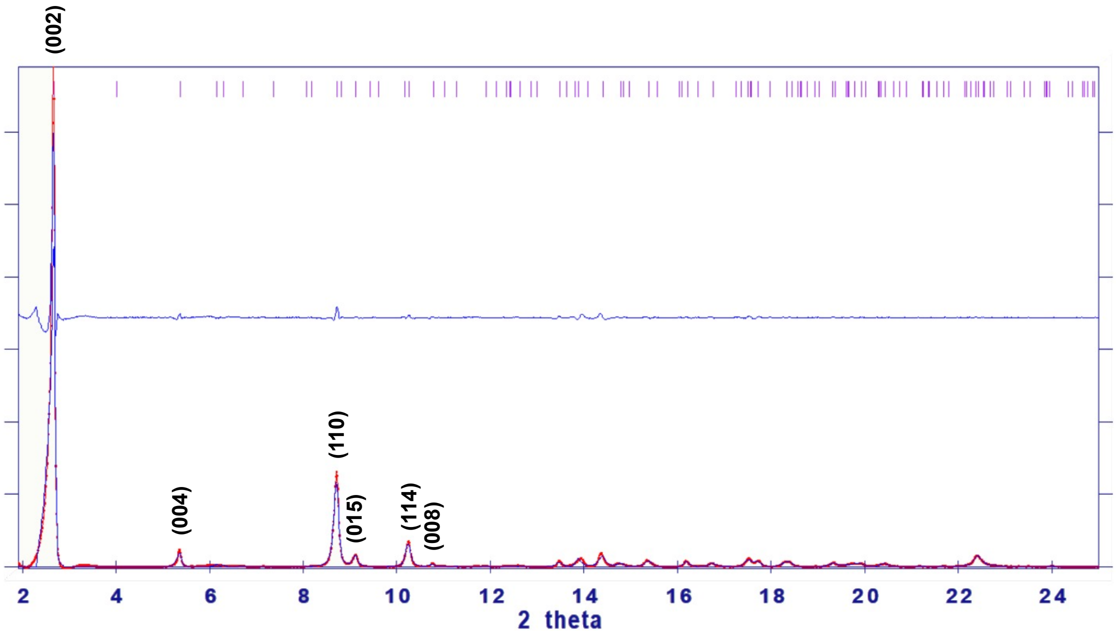

Figure S3. Pawley refinement of **Bet-COF-1** (a = b = 14.31 Å, c = 65.62 Å, R_{wp}= 19.28, and R_{exp}= 5.14). Red dots and blue trace are the experimental data and Pawley fit respectively, central blue trace is the difference between the experimental and the fitted data. All reflections for the Pawley obtained unit cell without symmetry are plotted as vertical purple lines. The complete refinement parameters are shown on **Table S2**.

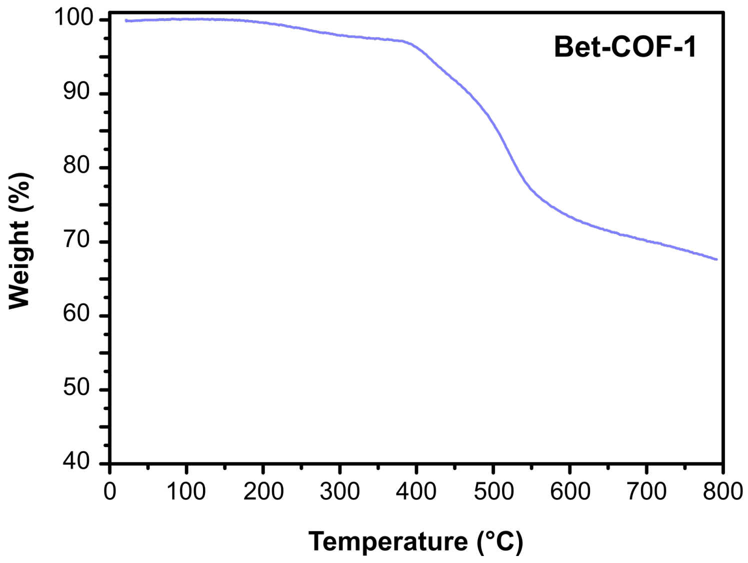

Figure S4. Thermogravimetric analysis of **Bet-COF-1** (after vacuum drying at 85 °C for 12 hours) performed at 10 °C min^{-1} under N_2.

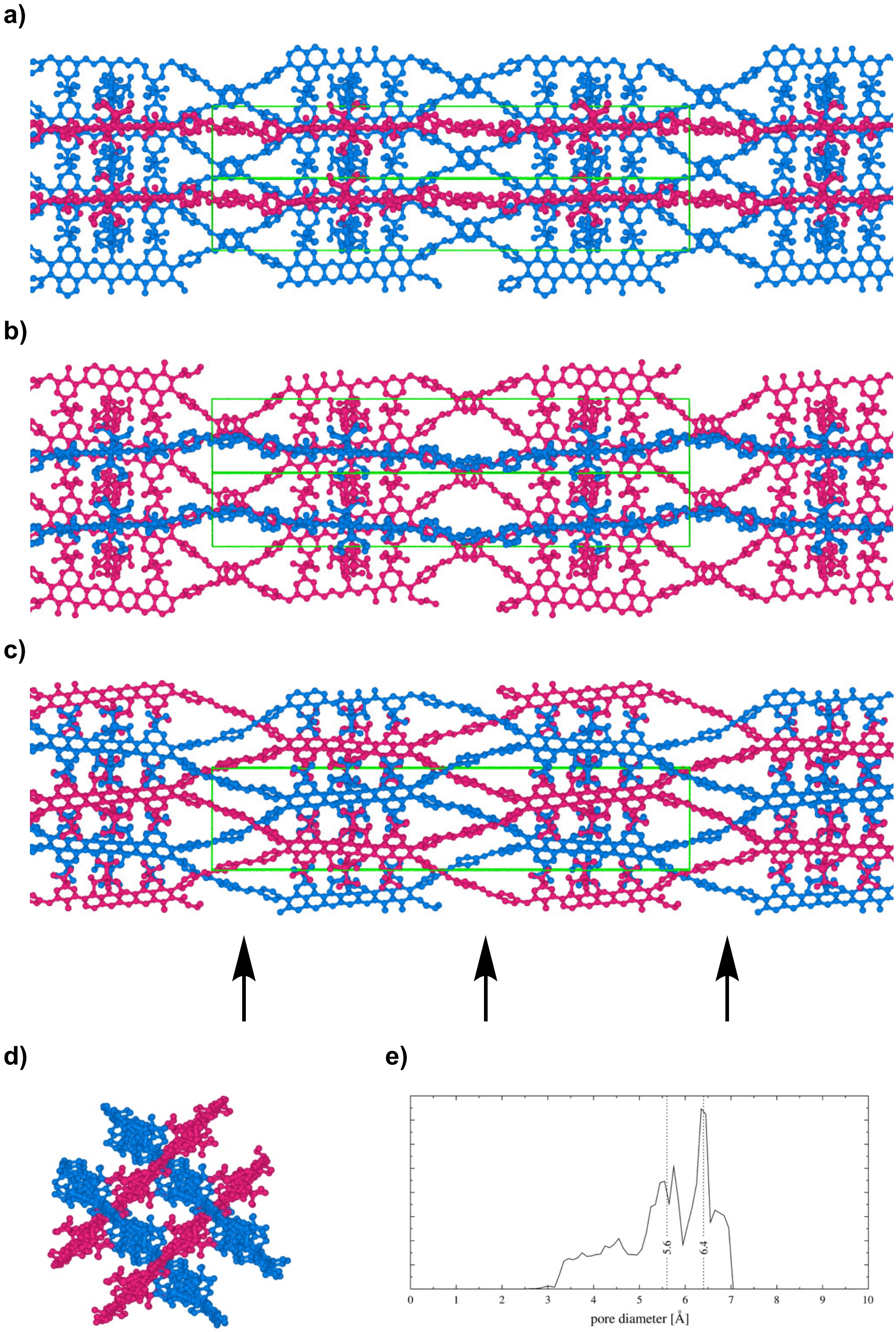

Figure S5. a-d) Two interpenetrated networks rendered in red and blue. Arrows indicate the space between of the *p*-phenylenediimine linkers that gives rise to the pores. e) Simulated pore size.

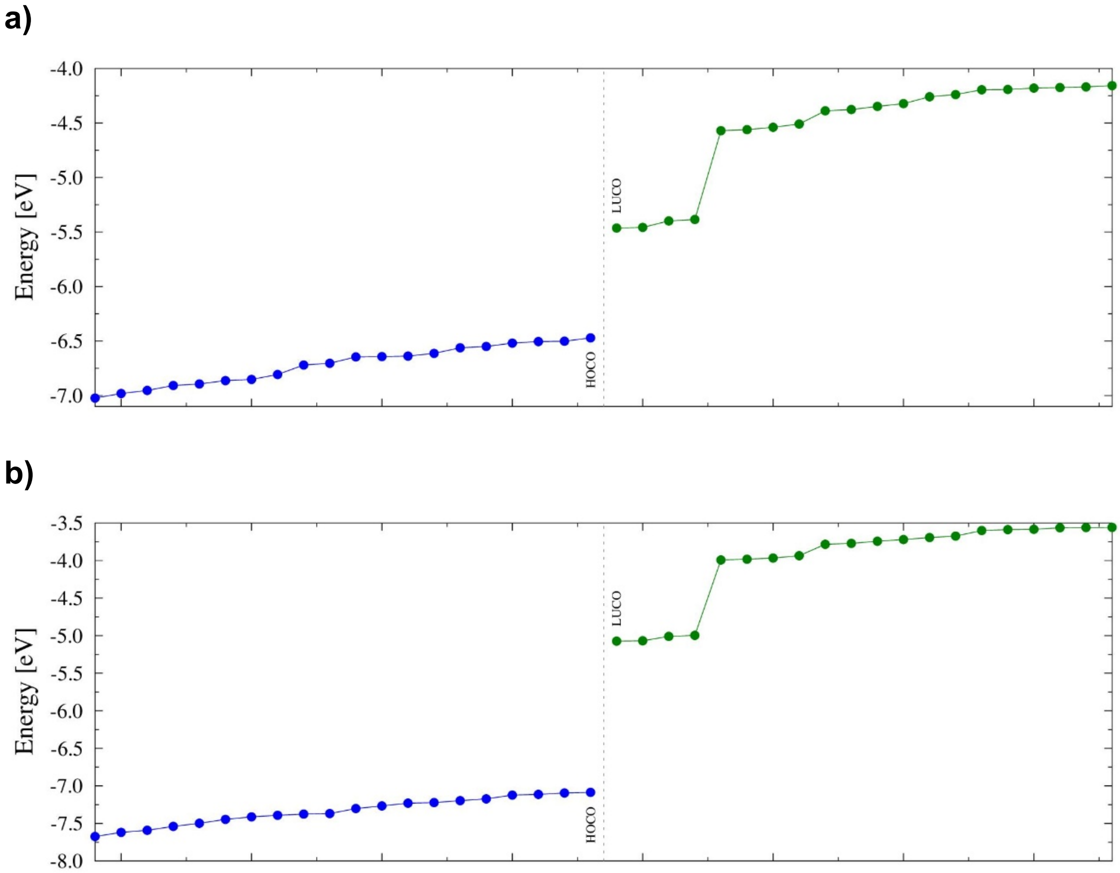

Figure S6. Energies of frontier orbitals of **Bet-COF-1** with (a) PBE/light Hamiltonian and (b) B3LYP/light Hamiltonian.

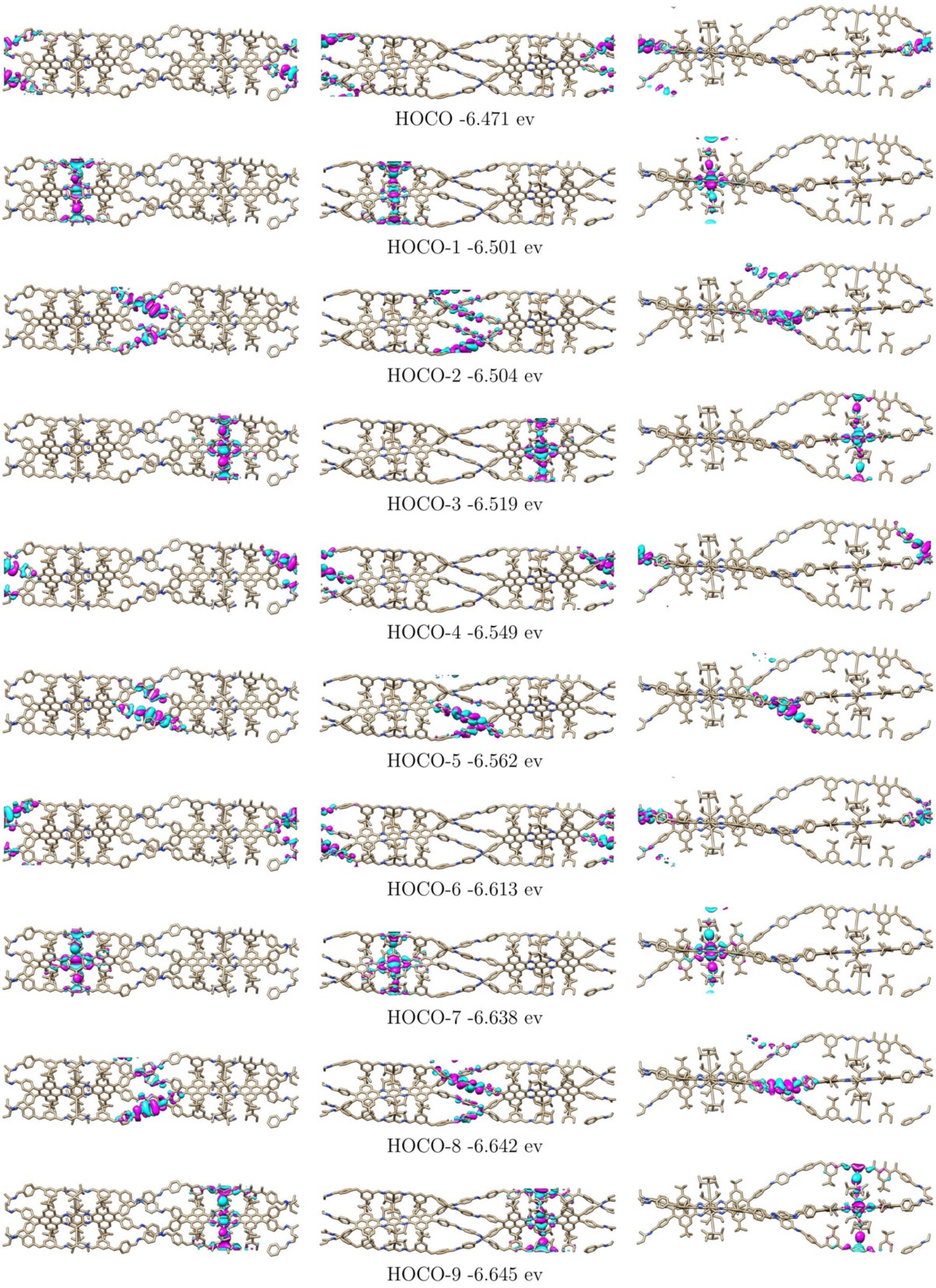

Figure S7. Highest Occupied Crystal Orbitals (HOCOs) and their energy for BET-COF-1 from three different viewpoints computed at PBE/light Hamiltonian level plotted with iso value of 0.02 e/Bohr (Γ point).

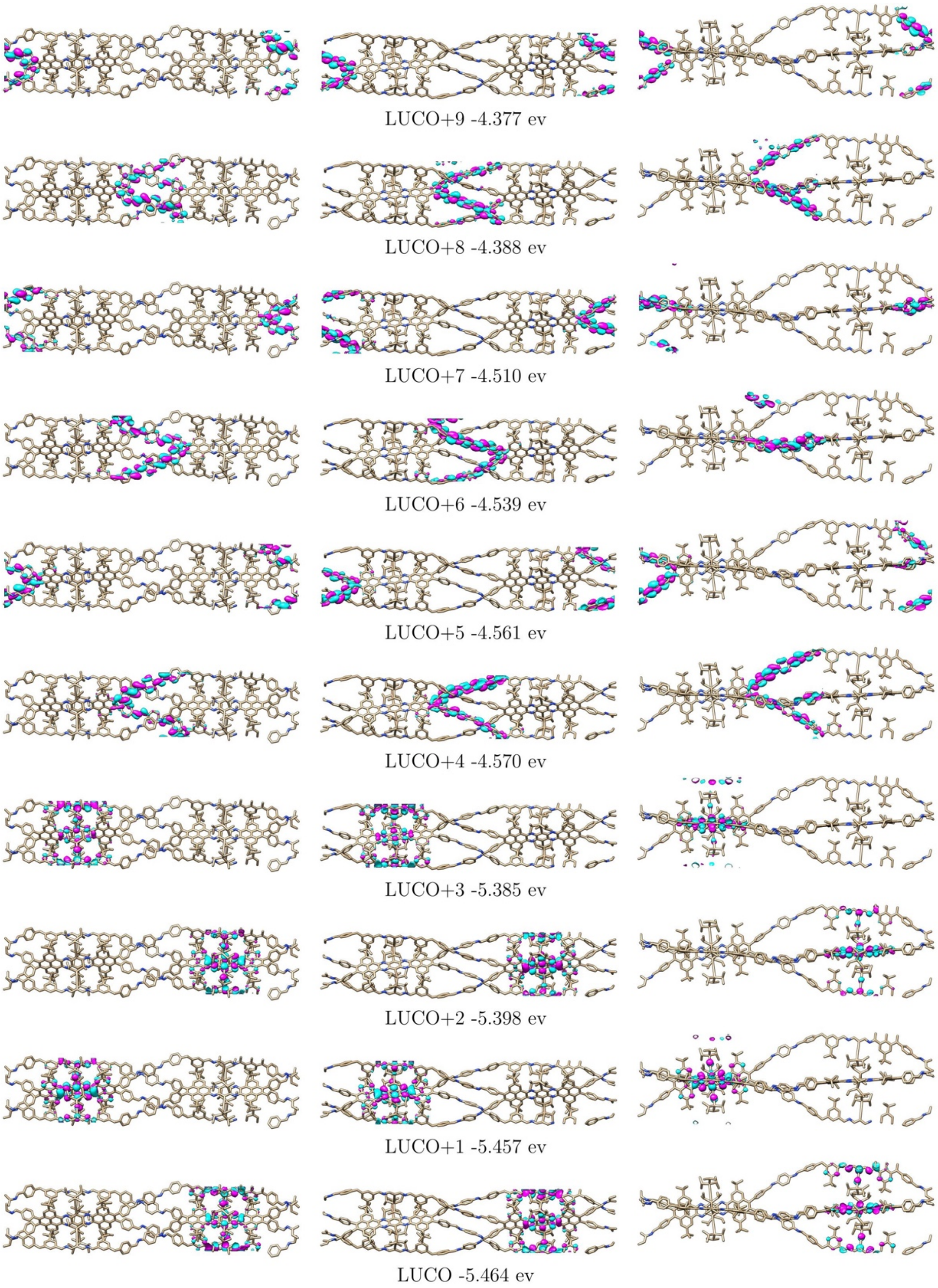

Figure S8. Lowest Unoccupied Crystal Orbitals (LUCOs) and their energy for BET-COF-1 from three different viewpoints computed at the PBE/light Hamiltonian level plotted with iso value of 0.02 e/Bohr (Γ point).

Table S1. MicroED Data collection, processing and refinement statistics

**Data collection**	
Excitation Voltage	200 kV
Wavelength (Å)	0.025079
Number of crystals	2
**Data processing** [a]	
Space group	Pnc2
Unit cell lengths ***a***, ***b***, ***c*** (Å)	65.63, 13.80, 14.13
Unit cell angles α, β, γ, (°)	90, 90, 90
Resolution (Å)	32.815-1.053 (1.08-1.05)
Number of reflections	27488
Unique reflections	9952
R_{obs} (%)	17.4 (144.8)
R_{meas} (%)	19.4 (170.8)
I/σ_I	3.63 (0.50)
$CC_{1/2}$ (%)	99.5 (39.4)
Completeness (%)	91.2 (78.8)
**Structure refinement**	
*R*1	0.1842
*wR*2	0.4622
GooF	1.201

[a] Values in parentheses represent the highest resolution shell

Table S2. Summary of the parameters obtained from the Pawley refinement.

Parameters	Refined Values
a, b [Å]	14.30891
c [Å]	65.62370
α, β, γ, (°)	90.000
Zero Point	-0.0008
Space Group	P4
R_{wp}	19.28
R_{exp}	5.14
χ^2	14.048

Experimental procedures

Reagents for synthesis were, if not otherwise specified, purchased from Aldrich, TCI or Acros Organics. Column chromatography was carried out using Silica gel 60 (40-60 μm) from Scharlab. Analytical thin layer chromatography (TLC) was done using aluminum sheets (20 x 20 cm) pre-coated with silica gel from Merck. UV-active compounds were detected with a UV-lamp from CAMAG at wavelength λ = 254 or 365 nm.

Bet-COF-1 was synthesized in a pre-scored 5 mL ampoule from Aldrich. For its synthesis and purification anhydrous mesitylene (98%), anhydrous dioxane (99.8 %), anhydrous dichloromethane (99.9%) and anhydrous acetone (99.8%) purchased from Acros Organics. THF was dried using an Innovative Pure Solve solvent purification system.

NMR spectra in solution were recorded on Bruker Avance 400 MHz pulsed Fourier transform NMR spectrometer at room temperature using partially deuterated solvents as internal standards. Chemical shifts (δ) are denoted in ppm. Multiplicities are denoted as follows: s = singlet, d = doublet, t = triplet, m = multiplet, br = broad.

Solid-State ^{1}H and ^{13}C CP/MAS NMR spectra were recorded on a Bruker Avance III 400 MHz NMR spectrometer at a MAS rate of 12 kHz and a CP contact time of 2 ms.

High-resolution mass spectra were recorded by Dr. Javier Calvo on UltrafleXtreme III MALDI tandem mass spectrometer (Bruker) in reflector acquisition operation mode and the samples were prepared in chloroform.

ATR-FTIR spectra were recorded on a Bruker ALPHA ATR-IR spectrometer.

Hydrolysis/digestion of **Bet-COF-1**. 2 mg of **Bet-COF-1** was dispersed in 0.5 mL of deuterated trifluoroacetic acid (TFA-d_1) in a vial. The dispersion was stirred at room temperature until the polymer was completely dissolved (approximately 48 h).

Thermogravimetric analysis was carried out on a TA Instruments Discovery system using a 10 ºC min^{-1} heating rate under a nitrogen flow.

Powder X-ray diffraction (PXRD) patterns for the optimization of the reaction conditions were collected by using a PHILIPS X'PERT PRO automatic diffractometer operating at 40 kV and 40 mA, in theta-theta configuration, secondary monochromator with Cu-Kα radiation (λ = 1.5418 Å) and a PIXcel solid state detector (active length in 2θ 3.347o). Data were collected from 1 to 50° 2θ (step size = 0.026 and time per step = 300 s, total time 40 min) at room temperature. A variable divergence slit, giving a constant 4.0 mm area of sample illumination, was used. The parameters of the final Pawley refinement are summarized in Table S2.

Electron Microscopy. All samples were prepared by dispersion in acetone and drop cast onto lacey carbon-coated copper TEM grids (Agar). High resolution SEM was performed on a JEOL 7100 F operated at 5 kV. High Resolution Transmission Electron Microscopy (HRTEM) analysis was performed on a JEOL2100F operating at 200 kV. The tilt series was taken using a Gatan 916 high tilt tomography holder, acquiring images at 1o tilt intervals and using a Gatan OneView electron detecting camera to ensure as low an electron dose as possible. The morphology of the samples was determined by taking low magnification (ca. x 30k mag) images from different regions of the specimen, and the nanoscale features were imaged using high resolution imaging (ca. x 100k mag).

MicroED Structure determination. For MicroED analysis, the powdered sample was applied to the surface of lacey carbon EM grids and excess sample was removed by gently tapping the tweezers holding the EM grids. Electron diffraction data were collected using standard small molecule MicroED procedures.[1-3] The samples were loaded into a Talos Arctica cryo-TEM operated at 200 kV and at cryogenic

temperature, equipped with a Falcon III direct electron detector. Continuous rotation MicroED data sets were collected crystals that showed high quality diffraction in the initial screening and MicroED data sets were processed using were indexed, integrated, and scaled using XDS.[4] Data from 2 crystals were merged and the structure of **Bet-COF-1** was determined by by direct methods in SHELXT[5] and refinement using SHELXL[6] The MicroED data collection, processing, and refinement statistics can be found in Table S1.

The porosity of the structure was evaluated by nitrogen sorption isotherms, measured at 77 K with a Micromeritics 3Flex apparatus. The sample was degassed in an Autosorb station at and 10^{-6} Torr at 100 °C prior to analysis. Surface area and volume values were calculated from nitrogen adsorption-desorption isotherms (77 K). Specific surface area (SA) was calculated by multi-point Brunauer-Emmett-Teller (BET) method.

Simulated surface areas were computed with Poreblazer 4.0[7] and Zeo++[8] with the default force-field and coefficients with a cubelet size of 0.1 Å for Poreblazer and a probe radius of 1.86 Å for Zeo++.

Solid-state UV-Vis-NIR electronic absorption spectra were recorded on a PerkinElmer Lambda 950 UV/VIS/NIR spectrophotometer by solid-state electronic diffuse reflectance.

Computational Models. The starting models were built from ED derived structures which being partial had to be completed with linker molecular pieces. Then, Tight Binding molecular dynamics were run with a static periodic cell. Selected structures from the dynamics trajectory were optimized at increasing levels of theory. The final models were obtained from Density Functional Theory (DFT) with the Fritz Haber Institute ab initio molecular simulations (FHI-aims) package.[9-11] For this, "light" numeric atomic orbitals, which approximately correspond to TZVP level of calculations were used with the PBE functional augmented with van der Waals Many Body Dispersion correction (MBD@rsSCS) unless otherwise stated.[12, 13] PBE

band structure and gap calculations were computed with an 2x2x2 grid of k-points, while the B3LYP hybrid functional, used for the band gap, was computed on a 2x2x2 k-point grid while the frontier orbitals were rendered in real space with Γ-point calculations.

FP-TRMC experiments were conducted for the sample on a quartz plate using the third harmonic generator (THG; 355 nm) of a Nd:YAG laser (Continuum Inc., Surelite II, 5–8 ns pulse duration, 10 Hz) as the excitation source (intensity I_0 = 9.1 × 10^{15} photons cm^{-2} $pulse^{-1}$). The frequency and power of microwave were ~9.1 GHz and 3 mW, respectively. The photoconductivity transient $\Delta\sigma$ was converted to the product of the quantum yield (φ) and the sum of charge carrier mobilities $\Sigma\mu$ (= $\mu_+ + \mu_-$) by the formula $\varphi\Sigma\mu = \Delta\sigma(eI_0F_{light})^{-1}$, where e and F_{light} are the unit charge of a single electron and a correction (or filling) factor, respectively.

Synthesis of 3

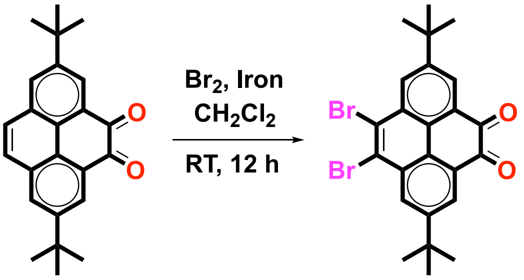

3

To a mixture of 2,7-di-*tert*-butylpyrene-4,5-dione (517 mg, 1.5 mmol), CH_2Cl_2 (50 mL) and 250 mg iron powder, a solution of Br_2 (959 mg, 6 mmol) in CH_2Cl_2 (25 mL) was added drop wise over 1 h at 0 °C with stirring. After the addition, the mixture was allowed to warm to room temperature and stirred overnight. Then, the mixture was poured into a large amount of ice water and stirred for 15 mins. The organic layer was washed successively with 10% aq. sodium thiosulfate, water and brine. The washed organic layer was then dried with sodium sulphate and concentrated. The residue was further washed with minimum amount of hexane and filtered to remove the byproducts. Then, the precipitate was collected and purified by column chromatography to give the desired compound **4** as an orange solid (670 mg, 89%).

^{1}H NMR ($CDCl_3$, 400 MHz, 298K) δ (ppm): 8.73 (d, J = 2.0 Hz, 2H), 8.61 (d, J = 2.0 Hz, 2H), 1.50 (s, 18H).

^{13}C-NMR ($CDCl_3$, 125 MHz, 298K) δ (ppm):180.30, 153.04, 133.41, 131.55, 129.92, 127.08,126.68, 35.99, 31.55.

MS (MALDI-TOF) (m/z): $[M+H]^+$ Calculated for $C_{24}H_{22}Br_2O_2$, 502.9768; experimental 503.0044.

Synthesis of 5

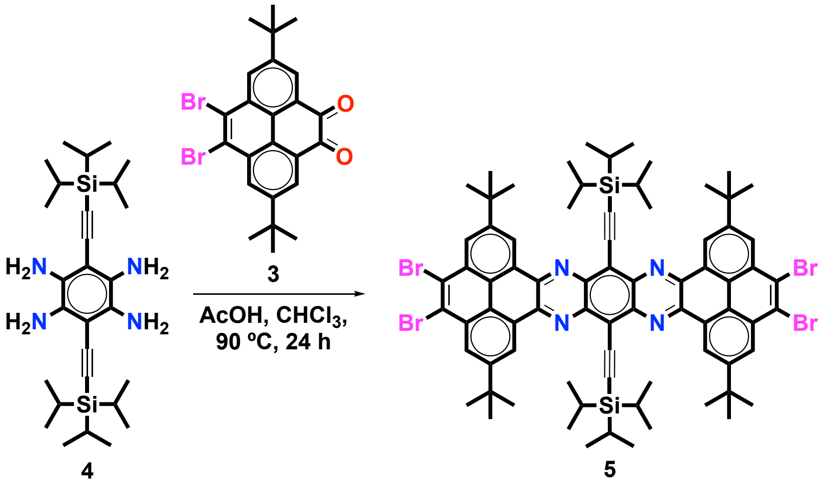

2,7-di-*tert*-butyl-4,5-dione-9,10-dibromopyrene (**3**) (1.00 g, 2.01 mmol) and 1,2,4,5-tetraamino-3,6-bis-[(triisopropylsilyl)ethynyl]-benzene (0.50 g, 1.00 mmol) were added to a 250 mL round bottom flask and degassed by pump-thaw cycles. Separately, acetic acid (150 mL) and chloroform (75 mL) were degassed by bubbling nitrogen for 20 minutes. Then, the degassed solvents were added to the reaction mixture under nitrogen and heated at 90 °C for 24 h. After cooling to room temperature, the reaction mixture was extracted with H_2O. After, the organic phase was dried over sodium sulfate and the solvent was evaporated. The residue was then subjected to chromatography (dichloromethane/hexane, 7 : 1) to afford **5** as a purple powder (411 mg, 29 %).

^{1}H NMR (400 MHz, CH_2Cl_2-d_2, 298 K, ppm): δ 9.91 (s, 4H), 8.95 (s, 4H), 1.72 (s, 36H), 1.69-1.53 (m, 6H), 1.39-1.37 (d, 36H).

^{13}C-NMR (Chloroform-d, 125 MHz, 298K) δ (ppm):151.81, 145.41, 143.00, 131.03, 129.82, 129.02, 127.29, 125.32, 125.14, 122.37, 110.53, 103.18, 36.24, 32.14, 19.52, 12.94.

MS (MALDI-TOF) (m/z): $[M+H]^+$ Calculated for $C_{76}H_{86}Br_4N_4Si_2$, 1427.3193; experimental 1427.3280.

ATR-FTIR (cm^{-1}): 2941, 2883, 1605, 1455, 1351, 1328, 1290, 1242, 1137, 967, 883, 769, 727, 679, 660, 442.

Synthesis of 1

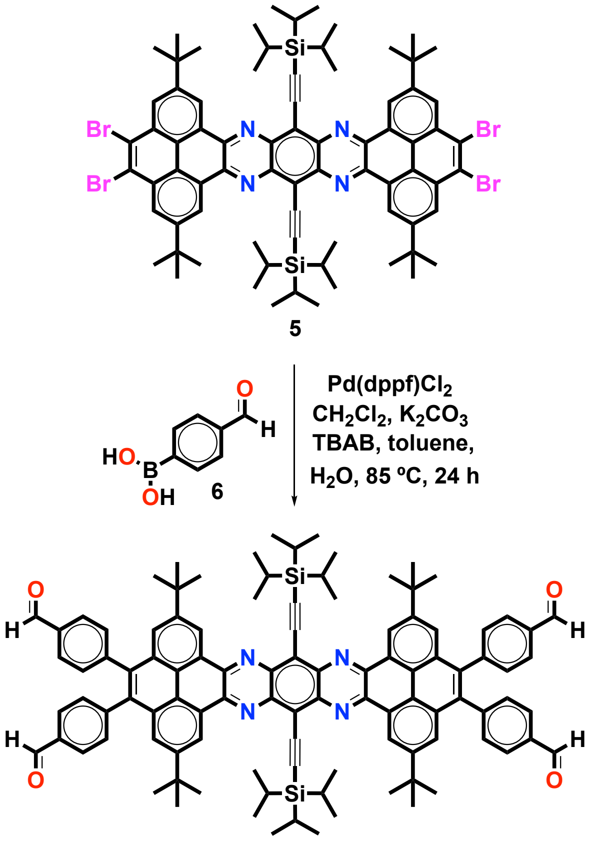

Compound **5** (150 mg, 0.11 mmol), 4-formylphenylboronic acid (157 mg, 1.05 mmol), tetrabutylammonium bromide (10 mg, 0.03 mmol), Pd(dppf)Cl_2·CH_2Cl_2 (26 mg, 0.03 mmol) and K_2CO_3 (218 mg, 1.58 mmol) were added to a 100 ml round bottom flask and degassed by pump-thaw cycles. Separately, toluene (45 mL) and water (15 mL) were degassed by bubbling nitrogen for 20 min. Then, the degassed solvents were added to the reaction mixture under nitrogen and heated at 85 °C for 24 h. After cooling to room temperature, the reaction mixture was extracted with H_2O. After, the organic phase was dried over sodium sulphate and the solvent was evaporated. The residue was then subjected to chromatography (dichloromethane) to afford **1** as a purple powder (51 mg, 32 %).

^{1}H NMR (400 MHz, CH_2Cl_2-d_2, 298 K, ppm): δ 10.04 (s, 4H), 9.88 (s, 4H), 7.96 (s, 4H), 7.89-7.87 (d, 8H), 7.55-7.53 (d, 8H), 1.67-1.59 (m, 6H), 1.51 (s, 36H), 1.39-1.37 (d, 36H).

^{13}C NMR (100 MHz, CH_2Cl_2-d_2, 298 K, ppm): δ 192.27, 150.68, 146.01, 145.94, 142.94, 137.11, 135.63, 132.30, 130.89, 130.07, 129.66, 126.90, 126.74, 125.03, 124.16, 35.86, 31.70, 19.25, 12.92.

MS (MALDI-TOF) (m/z): $[M+H]^+$ Calculated for $C_{104}H_{106}N_4O_4Si_2$ 1531.7822; experimental 1531.7768.

ATR-FTIR (cm^{-1}): 2951, 2883, 1703, 1604, 1460, 1364, 1300, 1234, 1206, 1137, 975, 882, 834, 807, 772, 735, 680, 418.

Synthesis of Bet-COF-1

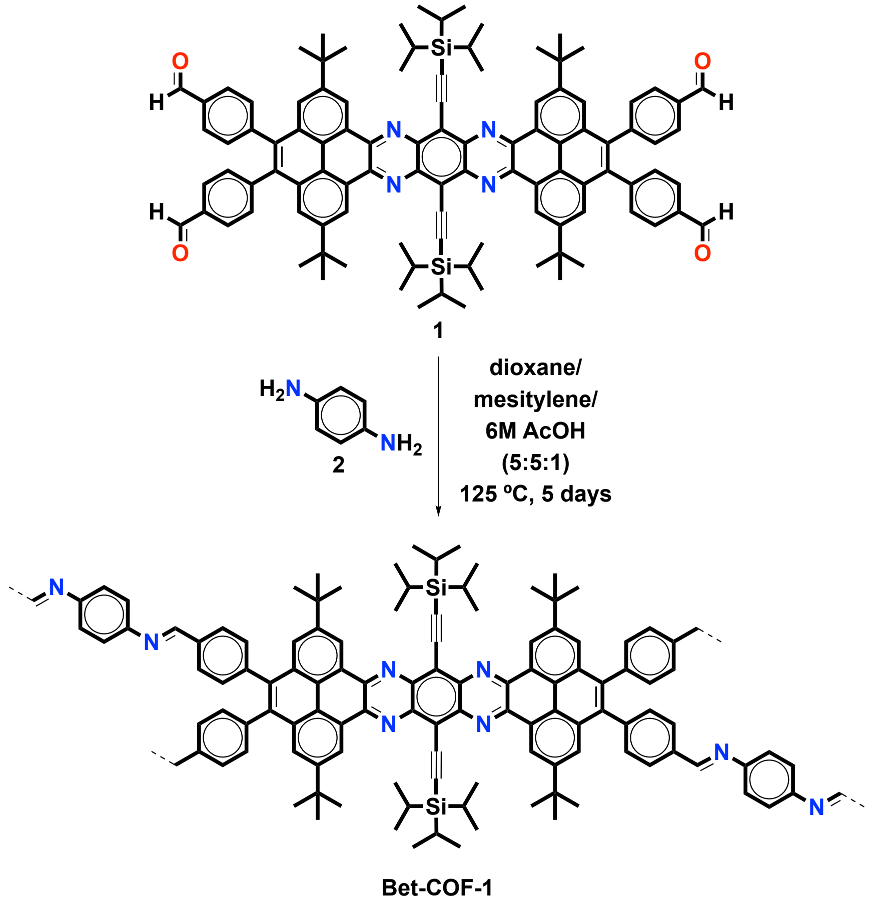

Compound **1** (15 mg, 9.8 µmol) and compound **2** (2.12 mg, 19.6 µmol) were sonicated in 0.5 mL of dioxane and 0.5 mL of mesitylene in a pre-scored 5 mL ampoule. Then, acetic acid 6M (aq.) (0.1 mL) was added to the mixture. The suspension was degassed by using three freeze-pump-thaw cycles. The ampoule was sealed off using flame and heated at 125 °C for 5 days. The purple precipitate was collected by filtration and washed five times with anhydrous tetrahydrofuran, dichloromethane, and acetone. The powder was dried at 85 °C under vacuum for 12 hours affording 14.6 mg of **Bet-COF-1** as a purple powder (89 % yield).

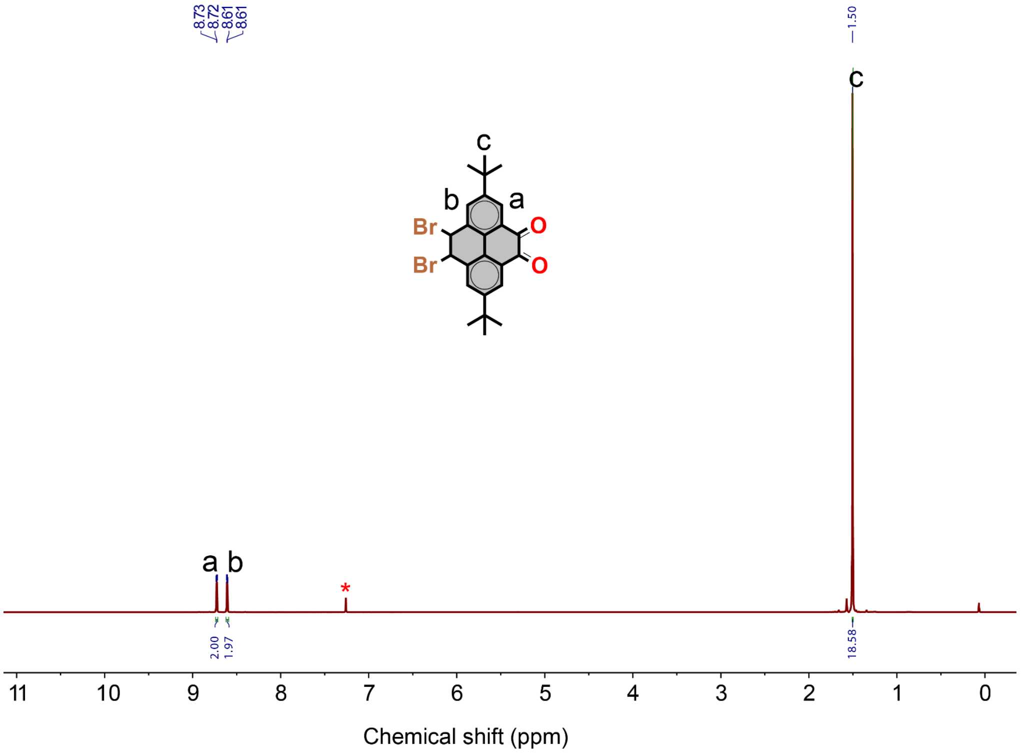

Figure S9. ^{1}H NMR spectrum of **3** (400 MHz $CDCl_3$, 298 K).

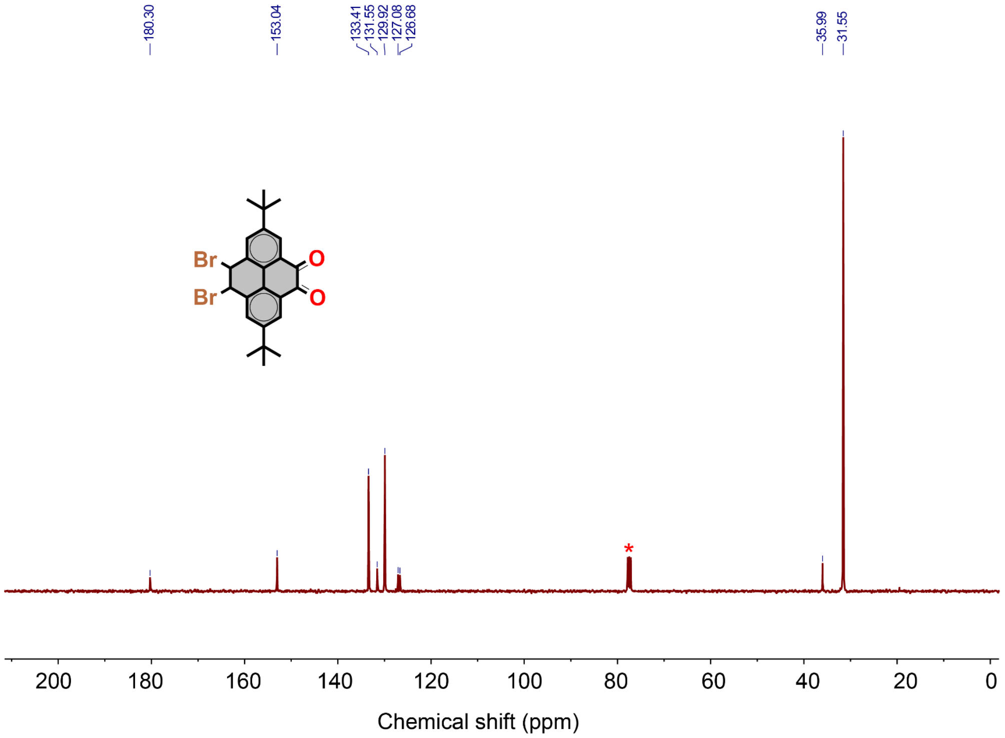

Figure S10. ^{1}C NMR spectrum of **3** (100 MHz $CDCl_3$, 298 K).

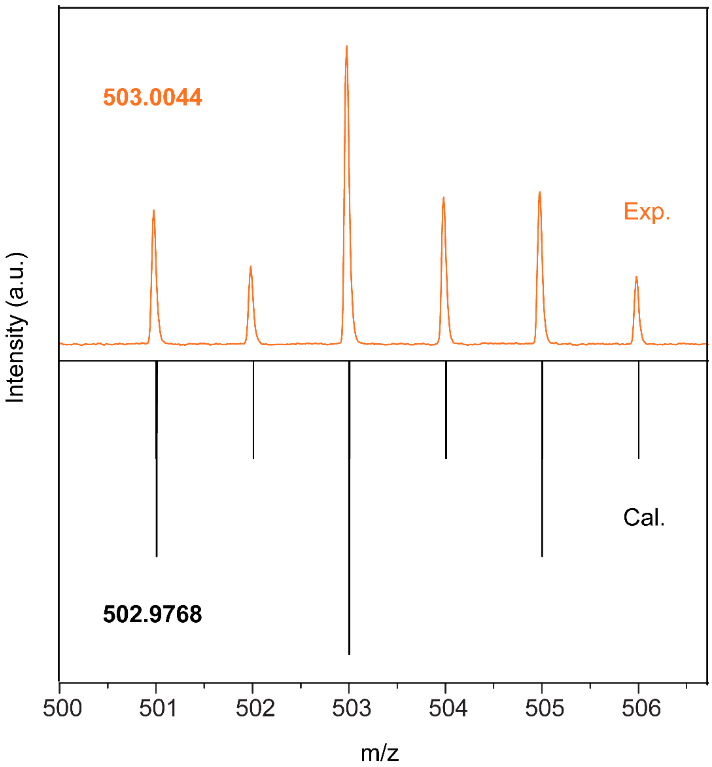

Figure S11. Experimental (top) and calculated (bottom) MALDI-TOF MS spectrum of **3**. Matrix: 10 mg/mL DCTB in THF, Adduct: $[M+H]^+$.

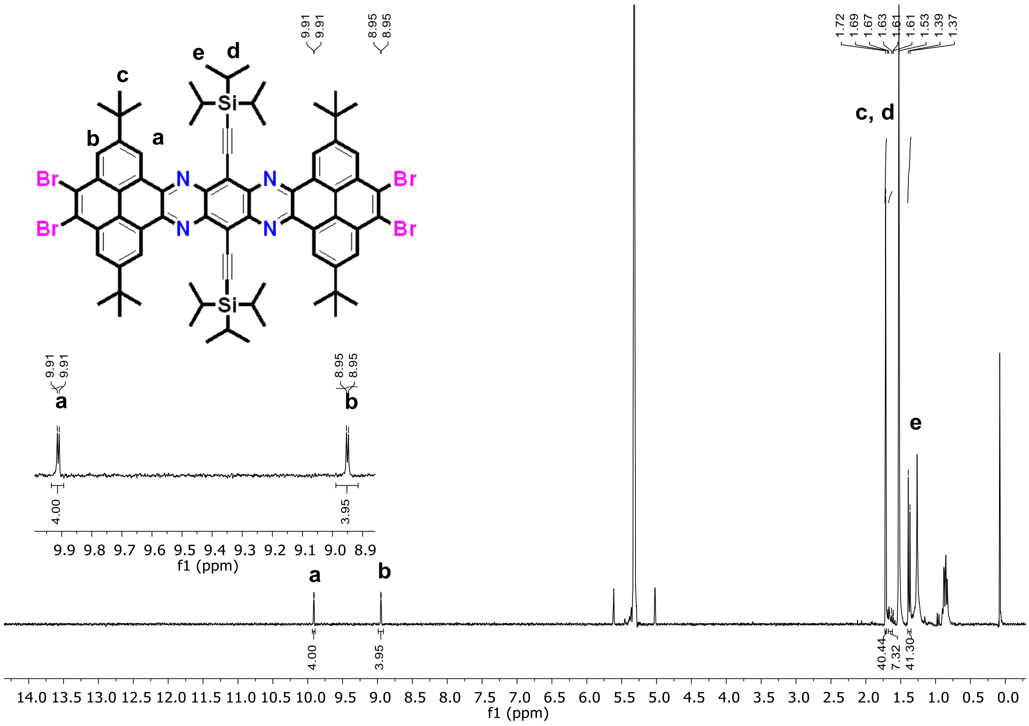

Figure S12. ^{1}H NMR spectrum of **5** (400 MHz CH_2Cl_2-d_2, 298 K).

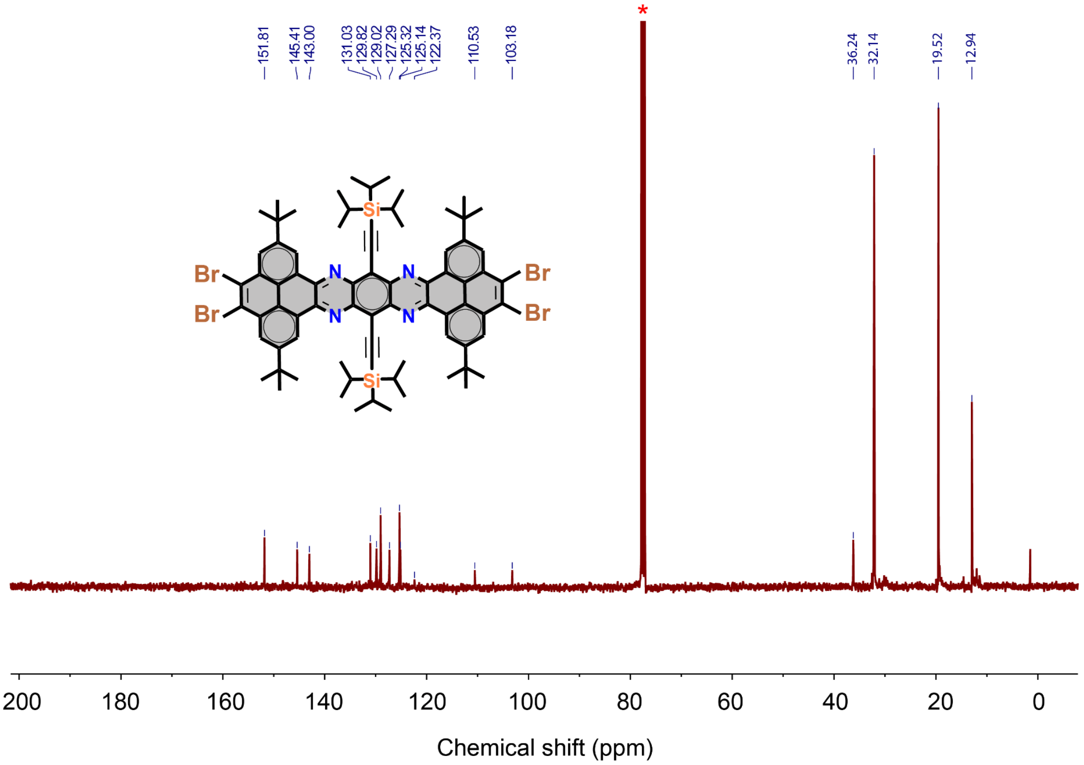

Figure S13. ^{13}C NMR spectrum of **1** (100 MHz, $CDCl_3$, 298 K).

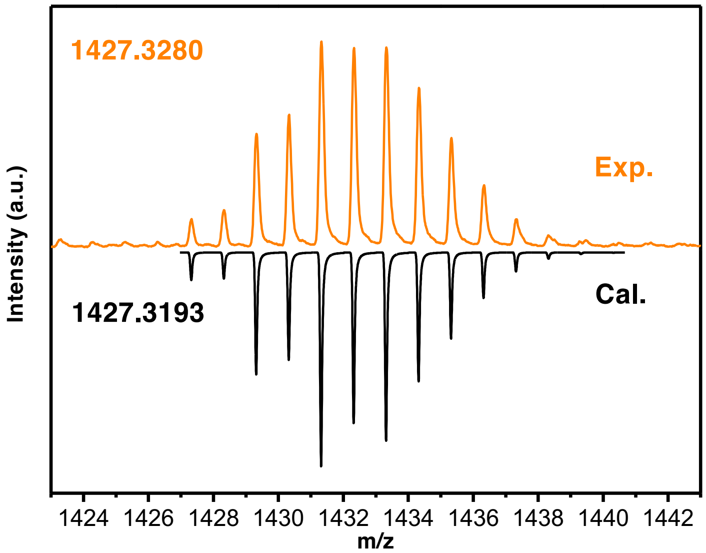

Figure S14. Experimental (top) and calculated (bottom) MALDI-TOF MS spectrum of **5**. Matrix: 10 mg/mL DCTB in THF, Adduct: $[M+H]^+$.

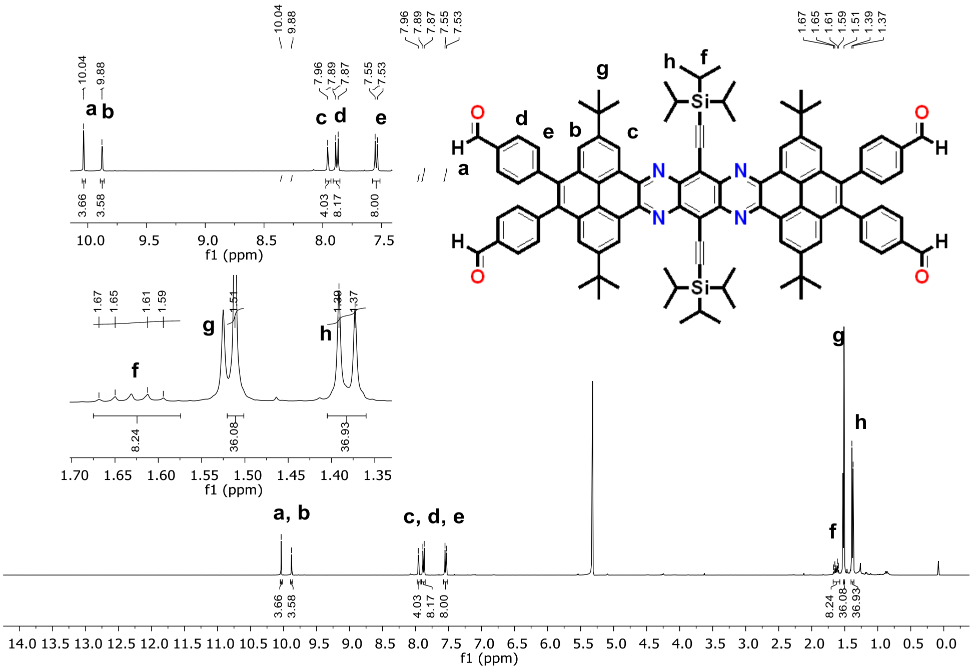

Figure S15. ^{1}H NMR spectrum of **1** (400 MHz, CH_2Cl_2-d_2, 298 K).

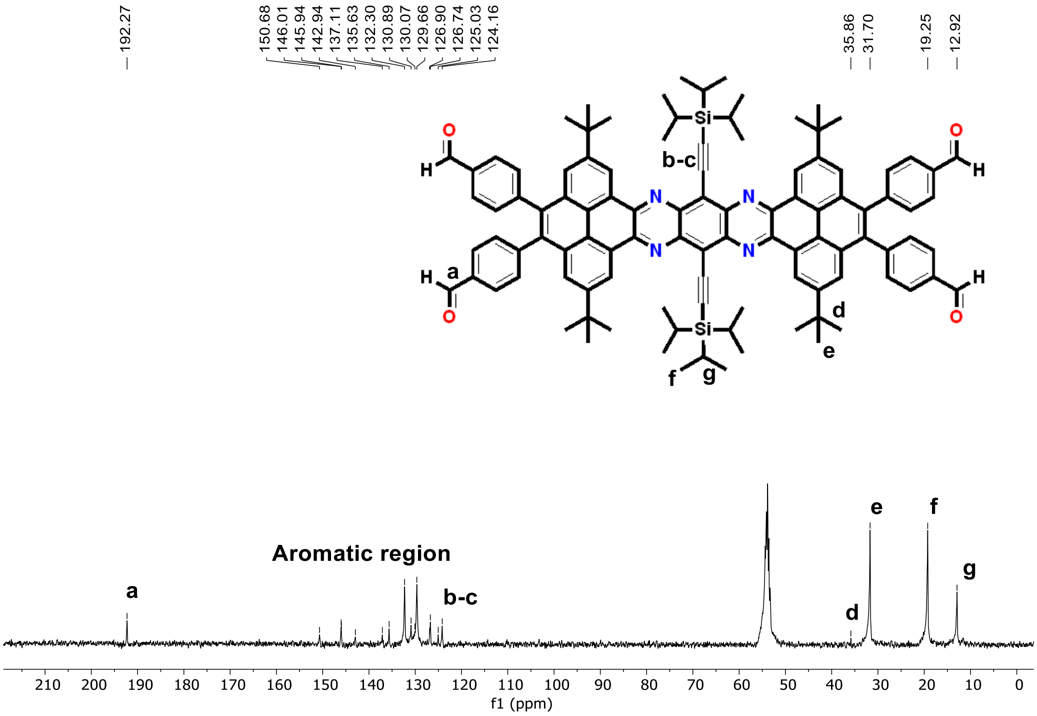

Figure S16. ^{13}C NMR spectrum of **1** (100 MHz, CH_2Cl_2-d_2, 298 K).

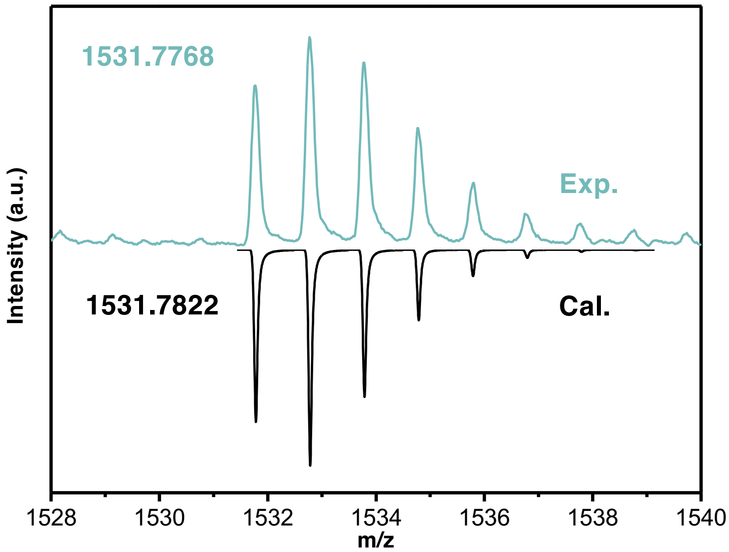

Figure S17. Experimental (top) and calculated (bottom) MALDI-TOF MS spectrum of **1**. Matrix: 10 mg/mL DCTB in THF, Adduct: $[M+H]^+$.

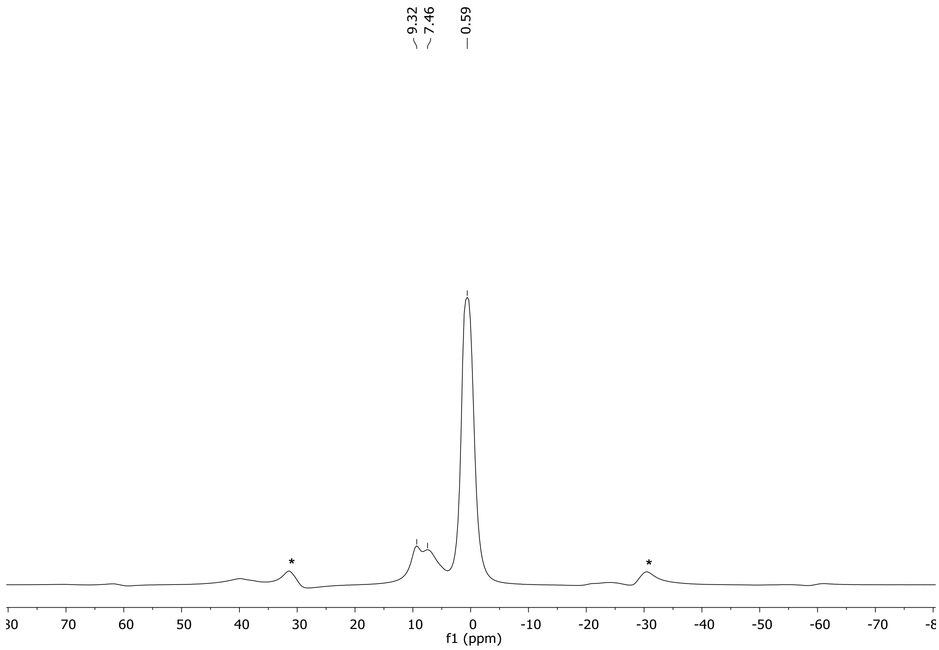

Figure S18. Solid-state ^1H NMR spectrum of **1** (* indicate spinning side bands).

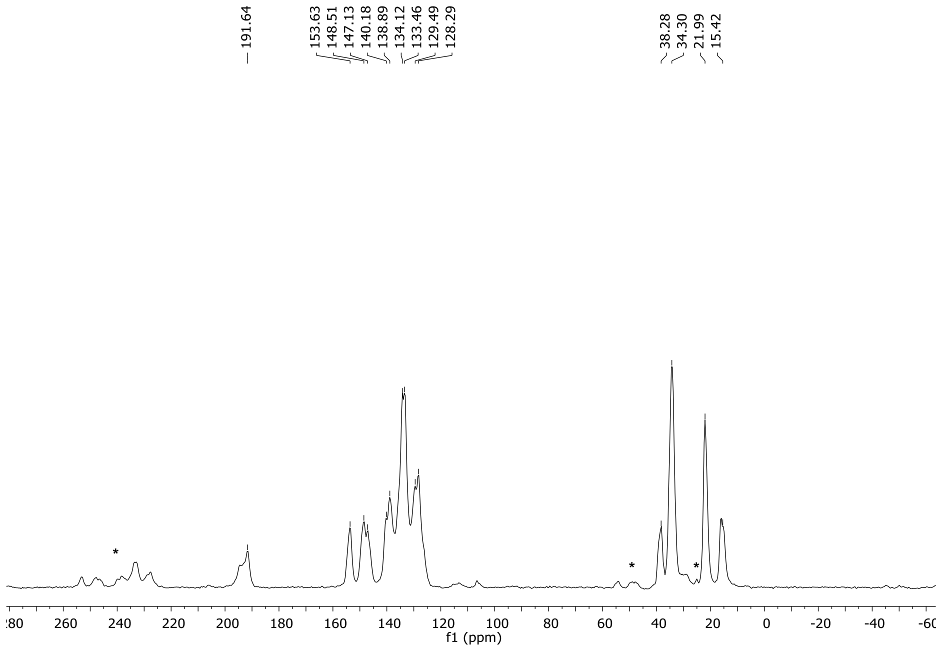

Figure S19. Solid-state ^{13}C NMR spectrum of **1** (* indicate spinning side bands).

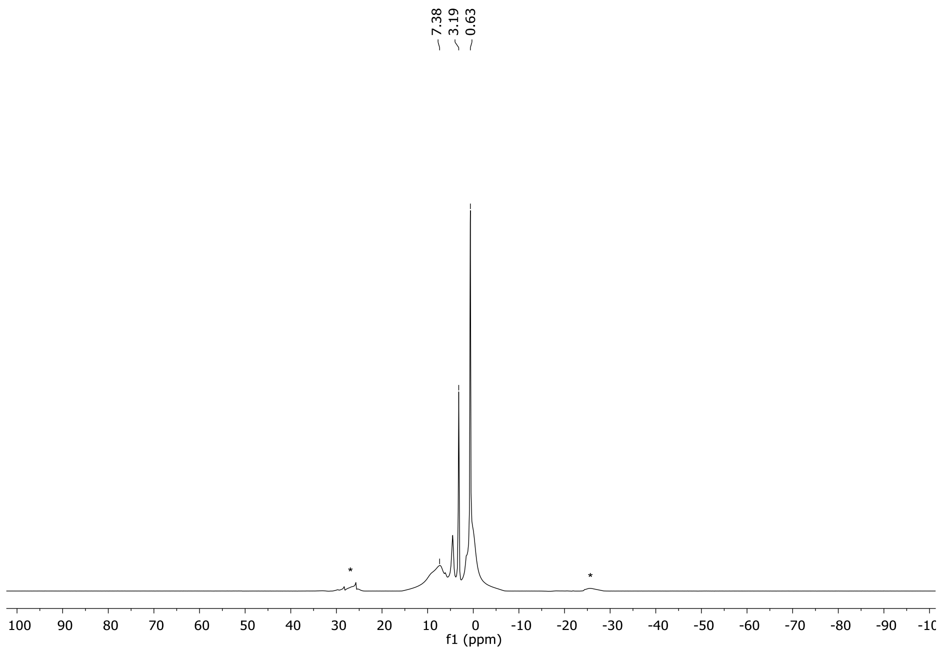

Figure S20. Solid-state ^{1}H NMR spectrum of **Bet-COF-1** (* indicate spinning side bands).

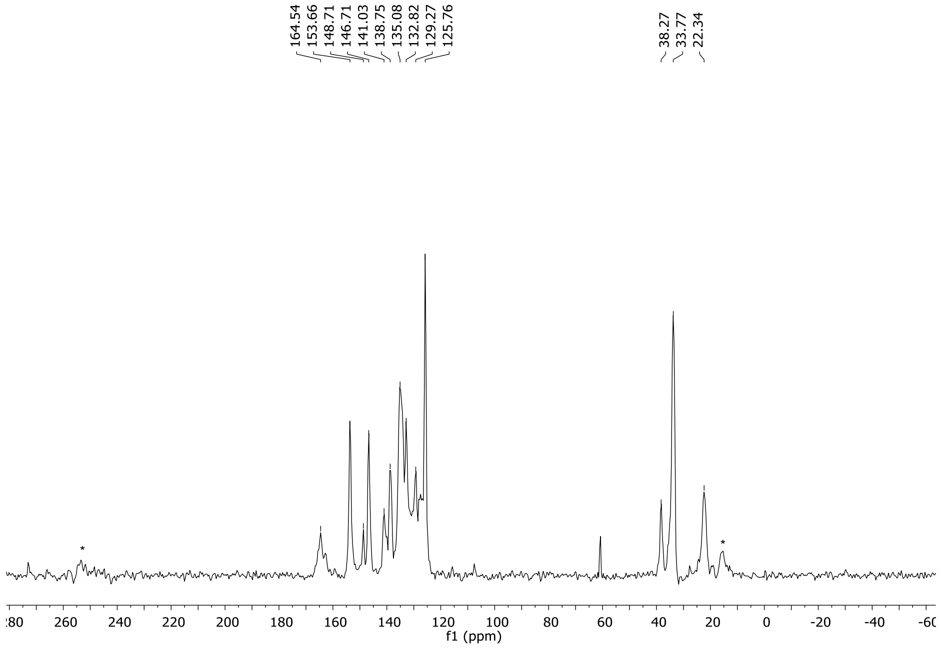

Figure S21. Solid-state ^{13}C NMR spectrum of **Bet-COF-1** (* indicate spinning side bands).

References

1. Haymaker, A.; Nannenga, B. L., Advances and applications of microcrystal electron diffraction (MicroED). *Curr. Opin. Struct. Biol.* **2024,** *84*, 102741.
2. Nannenga, B. L.; Shi, D.; Leslie, A. G. W.; Gonen, T., High-resolution structure determination by continuous-rotation data collection in MicroED. *Nat. Methods* **2014,** *11* (9), 927-930.
3. Jones, C. G.; Martynowycz, M. W.; Hattne, J.; Fulton, T. J.; Stoltz, B. M.; Rodriguez, J. A.; Nelson, H. M.; Gonen, T., The CryoEM Method MicroED as a Powerful Tool for Small Molecule Structure Determination. *ACS Cent. Sci.* **2018,** *4* (11), 1587-1592.
4. Kabsch, W., XDS. *Acta Crystallogr. D* **2010,** *66* (2), 125-132.
5. Sheldrick, G., SHELXT - Integrated space-group and crystal-structure determination. *Acta Crystallogr. A* **2015,** *71* (1), 3-8.
6. Sheldrick, G., A short history of SHELX. *Acta Crystallogr. A* **2008,** *64* (1), 112-122.
7. Sarkisov, L.; Harrison, A., Computational structure characterisation tools in application to ordered and disordered porous materials. *Mol. Simul.* **2011,** *37* (15), 1248-1257.
8. Willems, T. F.; Rycroft, C. H.; Kazi, M.; Meza, J. C.; Haranczyk, M., Algorithms and tools for high-throughput geometry-based analysis of crystalline porous materials. *Microporous Mesoporous Mater.* **2012,** *149* (1), 134-141.
9. Blum, V.; Gehrke, R.; Hanke, F.; Havu, P.; Havu, V.; Ren, X.; Reuter, K.; Scheffler, M., Ab initio molecular simulations with numeric atom-centered orbitals. *Comput. Phys. Commun.* **2009,** *180* (11), 2175-2196.
10. Marek, A.; Blum, V.; Johanni, R.; Havu, V.; Lang, B.; Auckenthaler, T.; Heinecke, A.; Bungartz, H. J.; Lederer, H., The ELPA library: scalable parallel eigenvalue solutions for electronic structure theory and computational science. *J. Condens. Matter Phys.* **2014,** *26* (21), 213201.
11. Yu, V. W.-z.; Corsetti, F.; García, A.; Huhn, W. P.; Jacquelin, M.; Jia, W.; Lange, B.; Lin, L.; Lu, J.; Mi, W.; Seifitokaldani, A.; Vázquez-Mayagoitia, Á.; Yang, C.; Yang, H.; Blum, V., ELSI: A unified software interface for Kohn–Sham electronic structure solvers. *Comput. Phys. Commun.* **2018,** *222*, 267-285.
12. Tkatchenko, A.; DiStasio, R. A.; Car, R.; Scheffler, M., Accurate and Efficient Method for Many-Body van der Waals Interactions. *Phys. Rev. Lett.* **2012,** *108* (23), 236402.
13. Ambrosetti, A.; Reilly, A. M.; DiStasio, R. A., Jr.; Tkatchenko, A., Long-range correlation energy calculated from coupled atomic response functions. *J. Chem. Phys.* **2014,** *140* (18), 18A508.
